# Results from the PROmoting Early Childhood Outside cluster randomized trial evaluating an outdoor play intervention in early childhood education centres

**DOI:** 10.1038/s41598-025-85397-1

**Published:** 2025-01-11

**Authors:** Rachel Ramsden, Dawn Mount, Yingyi Lin, Emily Fox, Susan Herrington, Janet Loebach, Adina Cox, Anita Bundy, Amber Fyfe-Johnson, Ellen Beate Hansen Sandseter, Michelle Stone, Mark S. Tremblay, Mariana Brussoni

**Affiliations:** 1 Department of Pediatrics, University of British Columbia, British Columbia Children’s Hospital Research Institute, F508 – 4480 Oak Street, Vancouver, BC V6H 3V4 Canada; 2 Human Early Learning Partnership, School of Population and Public Health, University of British Columbia, Vancouver, BC Canada; 3 Independent consultant, Seattle, WA USA; 4 School of Architecture and Landscape Architecture, University of British Columbia, Vancouver, BC Canada; 5 Department of Design + Environmental Analysis, Cornell University, Ithaca, NY USA; 6 Department of Landscape Architecture, Iowa State University College of Design, Ames, IA USA; 7 Department of Occupational Therapy, Colorado State University College of Health and Human Sciences, Fort Collins, CO USA; 8 Institute for Research and Education to Advance Community Health (IREACH), Elson S Floyd College of Medicine, Washington State University, Seattle, WA USA; 9 Department of Physical Activity and Health, Queen Maud University College of Early Childhood Education, Trondheim, Norway; 10 School of Health and Human Performance, Dalhousie University, Halifax, NS Canada; 11 Healthy Populations Institute, Dalhousie University, Halifax, NS Canada; 12 Healthy Active Living and Obesity Research Group, Children’s Hospital of Eastern Ontario Research Institute, Ottawa, ON Canada

**Keywords:** Human behaviour, Psychology and behaviour

## Abstract

Participation in outdoor play is beneficial for the health, well-being, and development of children. Early childhood education centers (ECECs) can provide equitable access to outdoor play. The PROmoting Early Childhood Outside (PRO-ECO) study is a pilot randomized trial that evaluates the PRO-ECO intervention on children’s outdoor play participation. The PRO-ECO intervention included four components: ECEC outdoor play policy; educator training; ECEC outdoor space modification; and parent engagement. This study included eight ECECs delivering licensed care to children (*n* = 217) aged 2.5 to 6 years in Greater Vancouver, British Columbia, Canada. Using a wait-list control cluster randomized trial design, ECECs were randomly allocated to either the intervention arm (*n* = 4) or the wait-list control arm (*n* = 4). Change in the proportion and diversity of observed outdoor play behaviour during scheduled outdoor time was measured. Outcome data were collected at baseline, 6-month follow-up, and 12-month follow-up. The intervention effect on children’s outdoor play participation was examined using logistic regression mixed effect models. Controlling for gender, weather and temperature, there were no changes in children’s outdoor play participation following implementation of the PRO-ECO intervention in the between-group analysis. Within-group comparisons also revealed no change in play participation following the PRO-ECO intervention, however, the intervention group showed a positive effect (OR = 1.28, 95% CI = 0.97, 1.70) in play participation 6 months after implementation of the intervention. The findings indicate that further analyses on child- and ECEC-level outcomes collected as part of the PRO-ECO study, including the diversity of children’s play, is required to effectively assess the impact of this intervention.

**Supplementary Information:**

The online version contains supplementary material available at 10.1038/s41598-025-85397-1.

## Introduction

Previous research has highlighted the importance of outdoor play for young children’s health, development and well-being^1–8^. Play is not just a leisure activity but a crucial mechanism through which children develop cognitive, social, and emotional skills^9–11^. Exposure to outdoor spaces, including nature, fosters creativity and imagination, providing a dynamic environment that stimulates learning and problem-solving skills^12,13^. Moreover, outdoor play encourages the development of motor skills, as children navigate various terrains and engage in activities like climbing, swinging, and balancing^6,14^. Socially, outdoor settings can offer opportunities for cooperative play, teamwork, and the development of essential social skills such as sharing and communication^6,15–17^. Children can also foster a connection with the natural world, instilling a sense of environmental awareness and responsibility from an early age^18,19^.

In many countries children’s participation in outdoor play has declined in recent years. This alarming trend is partially influenced by children’s increased use of technology, the changing landscape of neighbourhoods, and parental safety fears^8,20–22^. Participation in outdoor play in early childhood education centres (ECECs) has been shown to have unique benefits for children in the early years^23–25^. Over half of Canadian children under the age of 6 years are in a child care arrangement, with the majority of these children attending a child care centre or preschool setting^26^. Thus, ECECs are important settings for outdoor play, potentially providing novel opportunities that children may not experience in their home settings, and ensuring more equitable access to these opportunities^23^. ECECs provide diverse and multifaceted ways for children to engage in both structured and unstructured play, with activities ranging from organized games to spontaneous, imaginative play^27^. Structured play, often facilitated by educators, promotes skill-building, cooperative interactions, and a sense of routine^28–30^, while unstructured play allows children to explore their creativity, problem-solving abilities, and interpersonal skills independently^28,31–33^. Research underscores the role of the physical and social environments at ECECs in determining the quantity and quality of children’s outdoor play participation^34^.

ECECs across Canada are governed by federal, provincial or territorial, and municipal policies and regulations. Each province or territory has licensing regulations that govern how a child care centre must operate, focusing on the health and safety parameters. British Columbia’s (BC) provincial Child Care Licensing Regulations enforce a minimum of 6 m^2^ of outdoor play area for each child and a minimum of 60 min of outdoor active play per day^35^. Outside of these requirements, individual ECECs can determine the design and use of their outdoor space within the constraints of the safety-oriented licensing regulations. There are multiple challenges that ECEC environments experience to facilitating children’s outdoor play participation, including parent and educator perceptions of risk and safety^36,37^, the size and quality of the outdoor play space^38,39^, educator professional development opportunities^39^, and ECEC and regional policies and practices^40^. A multi-faceted approach that addresses multiple influences on children’s outdoor play is therefore required to create meaningful change within ECEC settings.

### Study objectives

Previous studies have looked at the influence of play-based interventions on children’s physical activity or active play behaviour^41–45^. While there is evidence in the literature on correlates of outdoor play at ECECs^39^, including educator training and environmental opportunities, there are limited studies that evaluate a multi-faceted outdoor play intervention administered in ECECs, especially using experimental study designs. The PROmoting Early Childhood Outside (PRO-ECO) pilot wait-list control cluster randomized trial implements and evaluates a comprehensive multi-faceted outdoor play intervention at eight ECECs in the Greater Vancouver region of BC, Canada. This paper provides the results of the primary outcome of the PRO-ECO study that seeks to assess the efficacy of the PRO-ECO intervention in increasing outdoor play participation in children aged 2.5 to 6 years at participating ECECs.

## Methods

**Fig. 1:**
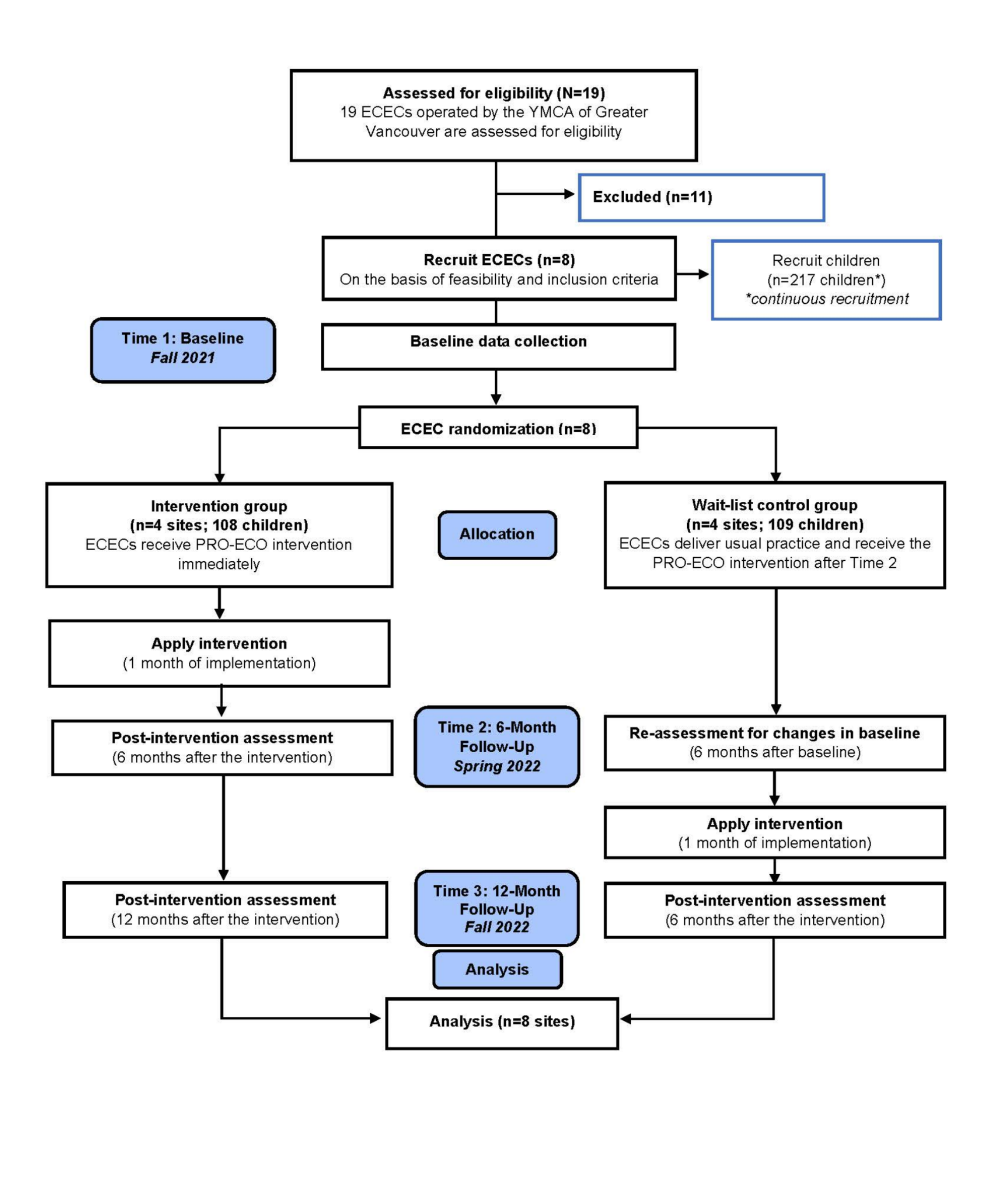
Wait-list control cluster randomized trial flow diagram: PROmoting Early Childhood Outside (PRO-ECO) pilot study.

The PRO-ECO study is a pilot wait-list control cluster randomized trial (trial registration: NCT05073380; 11/10/2021) that collected quantitative and qualitative data to assess the efficacy of the PRO-ECO outdoor play intervention. Data on outcome measures were collected at 3 time points: baseline (October – November 2021), 6-month follow-up (April–May 2022), and 12-month follow-up (October – December 2022). The wait-list control study design allowed for the assessment of short- and longer-term outcomes within the intervention group (Group 1) and short-term outcomes within the wait-list control group (Group 0) (Fig. 1). The methods outlined in this study are informed by the CONSORT (Consolidated Standards of Reporting Trials) statement for cluster randomized controlled trials^46^, the SPIRIT (Standard Protocol Items: Recommendations for Intervention Trials) statement for clinical trial protocols^47^ and the evaluation framework of the Medical Research Council^48^. The detailed PRO-ECO study protocol has been published^49^. Ethics certification was received from the University of British Columbia and the Children’s and Women’s Health Centre of British Columbia Research Ethics Board (H20-03912). All methods were performed in accordance with the relevant guidelines and regulations.

### PRO-ECO intervention

**Fig. 2:**
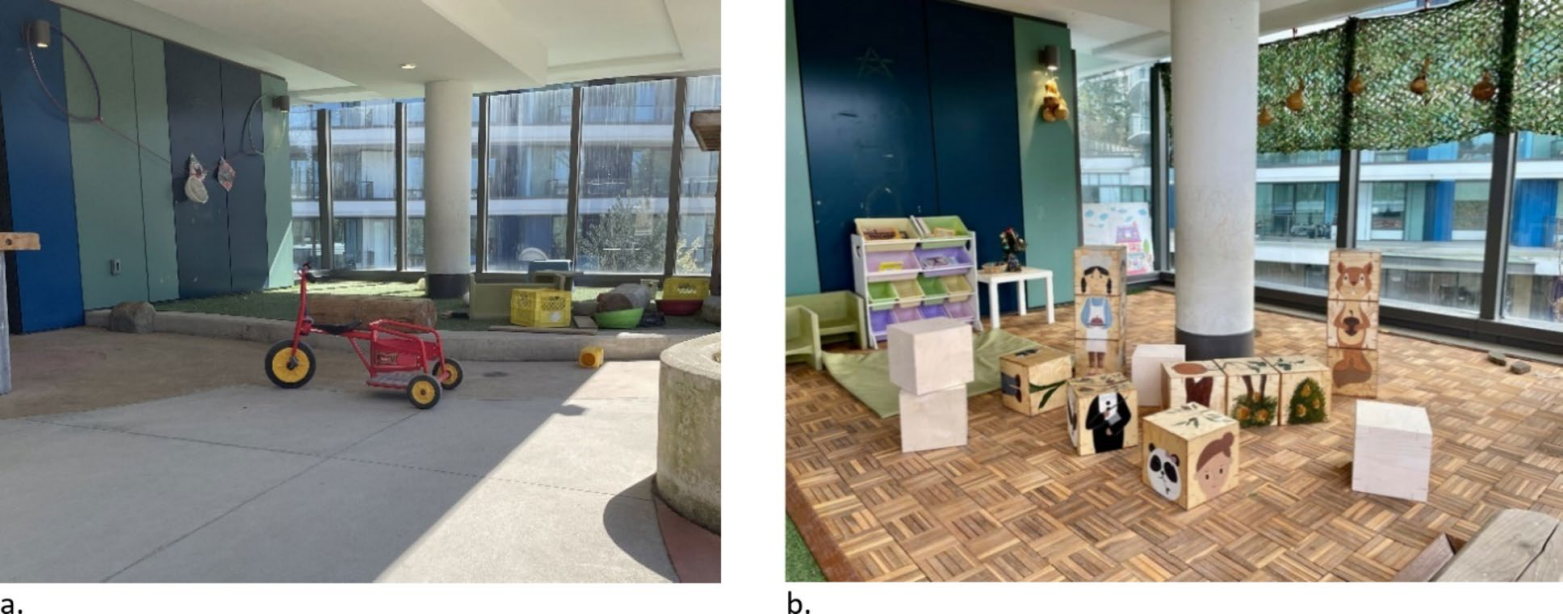
Centre A changes to surfacing materials. (**a**) Centre A Surfacing Materials Pre-Intervention. (**b**) Centre A Surfacsing Materials Post-Intervention.

**Fig. 3:**
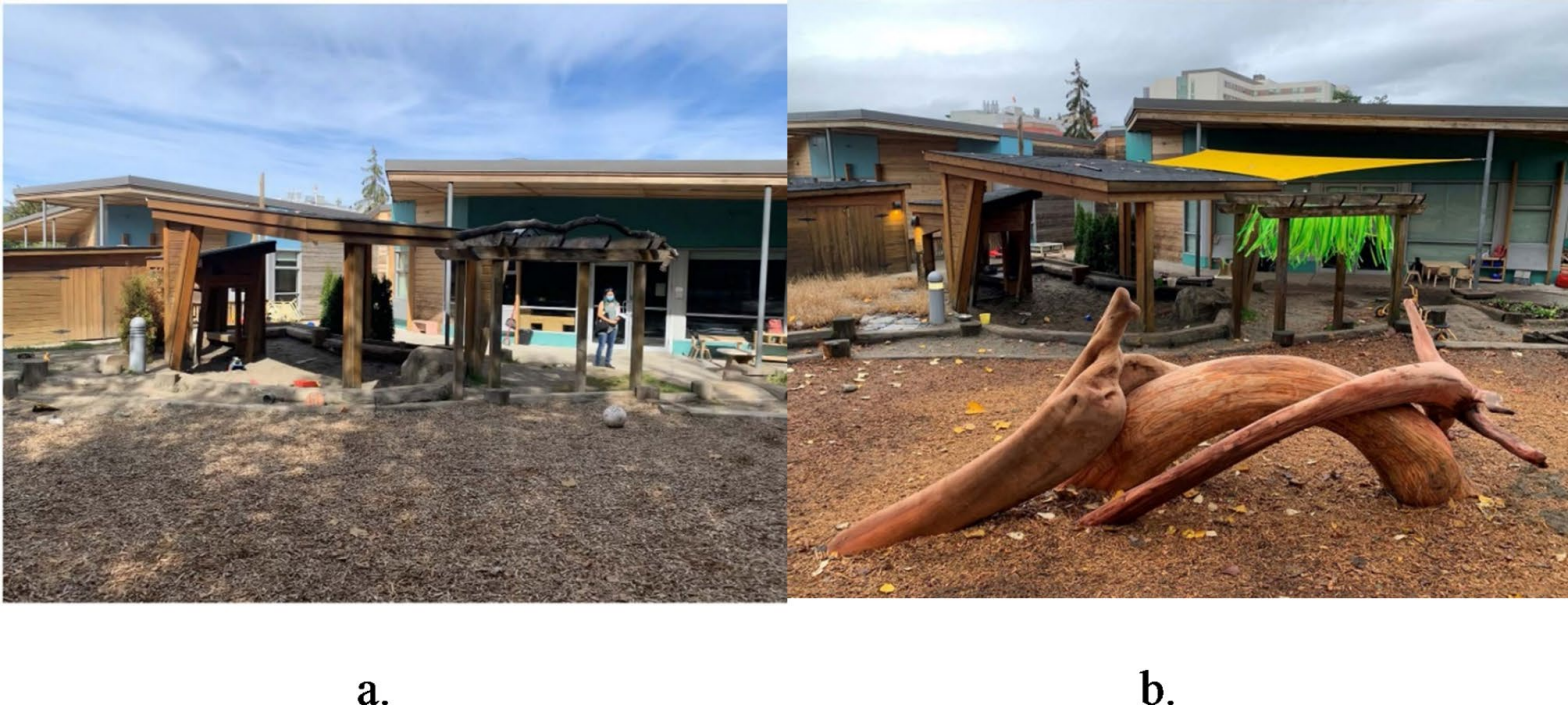
Centre C addition of climbing logs. (**a**) Centre B Open Space Pre-Intervention. (**b**) Centre B Open Space Post-Intervention.

**Fig. 4:**
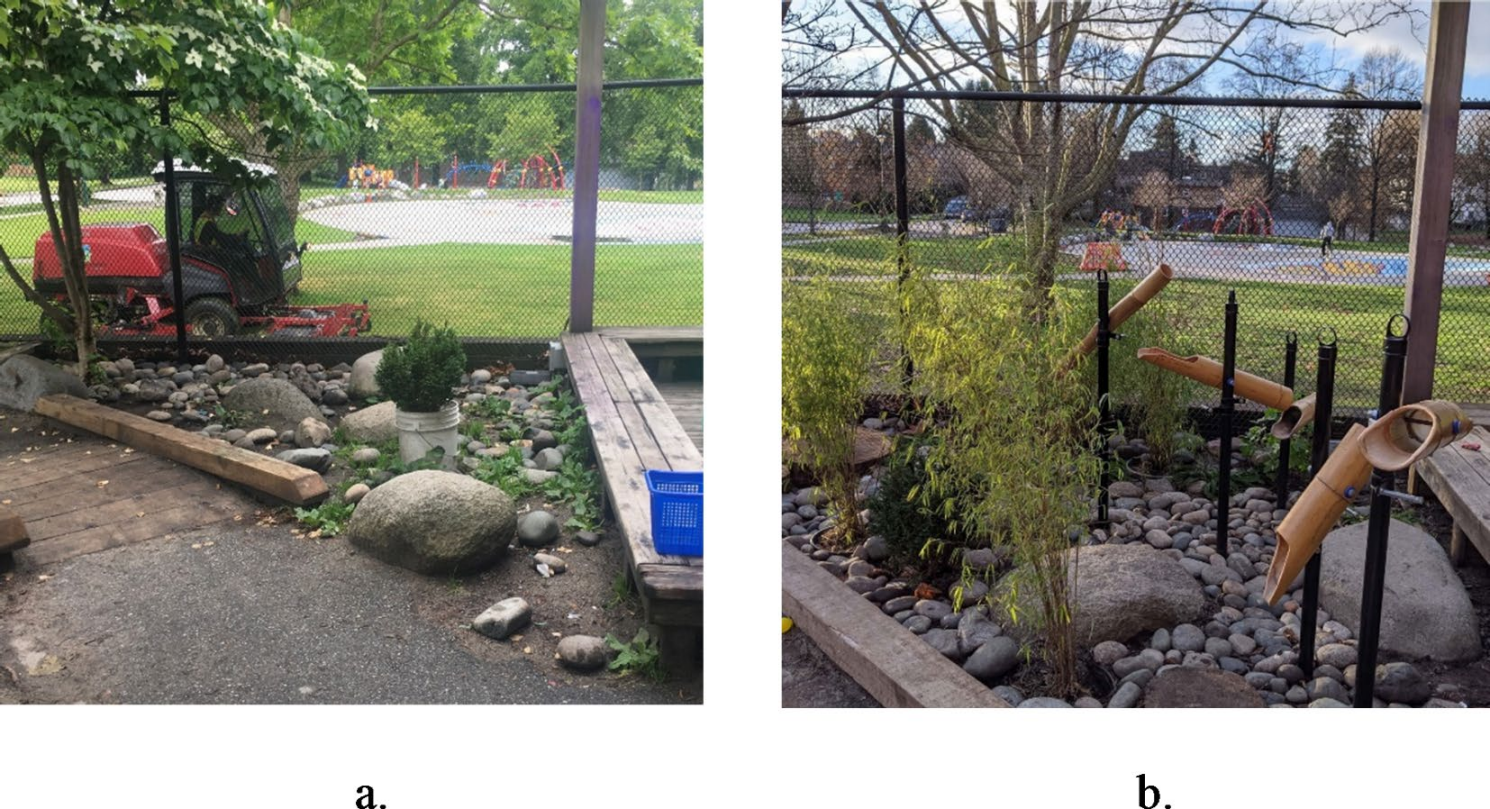
Centre D Addition of Rain Catchers. (**a**) Centre D Rock Area Pre-Intervention. (**b**) Centre A Rock Area Post-Intervention.

**Fig. 5:**
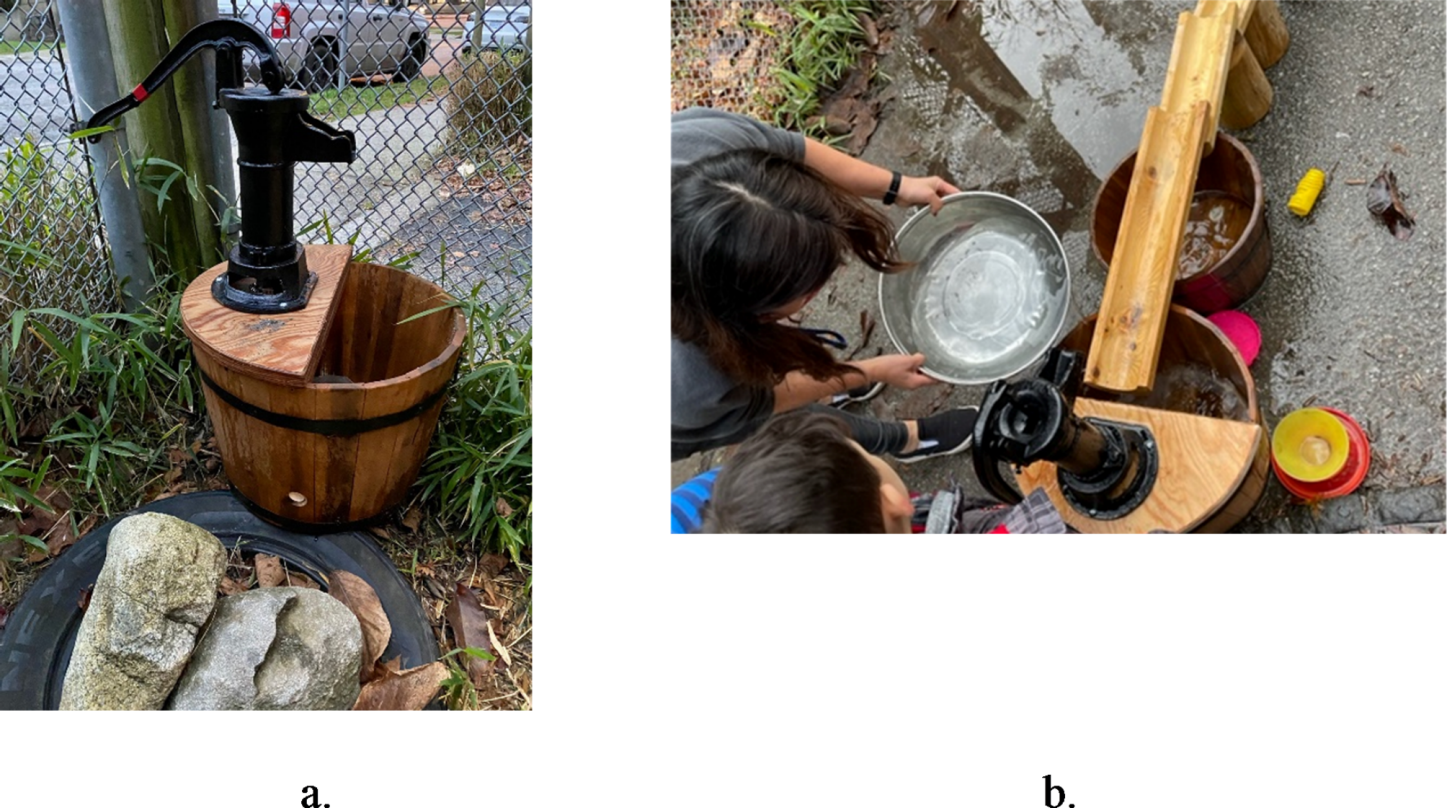
Centre G addition of moveable water pump post-intervention. (**a**) Centre G Moveable Water Pump. (**b**) Centre G Moveable Water Pump Used by Two Children.

The PRO-ECO intervention included four components: an ECEC outdoor play policy; ECE training; ECEC outdoor space modification; and parent engagement (further details in Table 1). The PRO-ECO intervention was also tailored to individual ECEC sites based on their identified needs. Following baseline data collection, the intervention was further refined to provide site-specific adjustments, including specific materials in the built environment design modification or targeted follow-up training and mentorship. The individualization of the intervention to each ECEC was performed following initial analyses of baseline data identifying how and where children play, and through focus groups with ECEs. Full details on the process followed to develop and undertake the PRO-ECO intervention has been previously published^50^ and a sample of important space modifications that were completed can be seen in Figs. 2, 3, 4 and 5 and Supplementary Material 1. In addition to the four key intervention components, the study team secured loose parts, which included shells, pinecones, fabric, water piping and miscellaneous kitchen items (see Supplementary Material 1), for each ECEC and donated rain gear items for children on an as-needed basis.

Stratified randomization of participating ECECs occurred prior to intervention delivery. The percentage of families enrolled in the BC Affordable Child Care Benefit (< 100% or 100%), a government subsidy to support eligible families with the cost of child care^51^, and type of facility (above-grade or at-grade) were used to stratify the 8 ECECs. Within each stratum, block randomization was applied to assign each center to the intervention arm (Group 1) or the wait-list control arm (Group 0) using Research^52^. ECECs randomly assigned to Group 1 received the PRO-ECO intervention immediately following completion of Time 1 baseline data collection. ECECs randomly assigned to Group 0 received the PRO-ECO intervention after Time 2 data collection occurred at 6 months from baseline (see Fig. 1). The research trial coordinator (RR) completed the randomization of ECECs. The research project coordinator (DM) recruited and enrolled participants. The research team was blinded to the intervention status of each ECEC during baseline data collection and at the analysis stage.

**Table 1:** PROmoting early Childhood outside (PRO-ECO) outdoor play intervention components.

Intervention component	Intervention activity	Universal vs. tailored to ECEC^a^
ECEC outdoor play policy	Identification by the YMCA^b^ management team of the organization’s values and vision regarding outdoor play, including priorities and principles. The values and vision were disseminated to the ECECs. These outdoor play values were used by each ECEC to review existing policies, procedures and practices (e.g., supervision plans, parent handbooks) for alignment and to modify accordingly. Implementation of values and modified policies across all participating ECECs.	Universal
ECE training	A series of training sessions and opportunities for ECEs^c^, including: Training workshop that included content on the importance of outdoor risky play, methods for risk-benefit assessment, and encouragement of the use of loose parts. This training was offered to Group 1 as an all-day in person workshop. Group 0 received the training as an online 4-hour workshop due to COVID-19-related restrictions. An in-person all-day workshop was offered prior to Time 3 data collection for all ECEs who were new and had not already received the training. This training was developed and provided by the YMCA of Southwestern Ontario and is being rolled out to YMCA ECECs across Canada. The training was designed to include on-going mentorship of each ECEC through, for example, discussions at staff meetings. ECE outdoor play web-based training tool and resource, OutsidePlay.org(72), was available for asynchronous learning.	Universal
ECEC outdoor space modification	Outdoor space modification for each ECEC as follows^50^: Design plans for each center were developed by 14 University of British Columbia School of Architecture and Landscape Architecture (SALA) graduate students in a design studio. These were based on the Seven Cs design principles^d^^53^. Graduate students designed modifications to the built environment in consultation with ECECs. SALA graduate students and PRO-ECO research team implemented modifications. A budget of CAD $4,000 for general expenses and CAD $2,000 for shade-related interventions was available for each site. Further details on outdoor space modifications implemented in each ECEC can be seen in Supplementary Material 1.	Tailored
Parent engagement	Parent engagement materials and events provided to increase knowledge of the importance of outdoor play and encourage parent involvement in implementing the outdoor space modification. These varied by ECECs to suit their needs, and included posters in ECECs, distributing infographics to parents, rock-painting events, plant voting, and other activities as chosen by ECECs, with support from the PRO-ECO research team. Pedagogical narration of children’s outdoor play experiences and learning were posted by ECEs on the internal ECEC organization mobile app for access by parents.	Tailored

^a^ECEC: early childhood education center.

^b^YMCA: YMCA of Greater Vancouver.

^c^ECE: early childhood educator.

^d^The Seven C’s design principles include character, context, connectivity, change, chance, clarity and challenge.

### Study sample

This is a pilot study and the sample size was based on the feasibility of administering the PRO-ECO intervention to multiple ECECs. The PRO-ECO study included 8 ECECs delivering licensed, full-day, group care to children aged 2.5 to 6 years and operated by the YMCA of Greater Vancouver (YMCA GV). The YMCA GV is a not-for-profit organization that delivers child care services, as well as other family and community services. The participating ECECs were located in 3 different cities within the Greater Vancouver region (Canada) and receive government funding to support the day-to-day costs of operation, enhance ECE wages and reduce fees. All participating centres had outdoor spaces that were directly adjacent to their indoor space, but ranged in size, available play affordances and ground surfaces present (Table 2). In addition to fixed play affordances, participating ECECs had access to loose parts, including sand toys, cooking utensils, planks and stumps, and gardening tools, as well as wheeled toys such as tricycles and scooters. Each ECEC was licensed for 25 children within the participating age group (2.5–6 years) and the examined outdoor play space was dedicated for the licensed capacity and program type. Further detail on each of the participating ECECs is depicted in photos available as Supplementary Material 1. Characteristics in Table 2 that have been identified as being altered or added as part of the outdoor space modification component of the intervention are outlined in Figs. 2, 3, 4 and 5. Additional photos and further details of the outdoor space modifications that occurred for each ECEC are outlined in Supplementary Material 1.

### Study recruitment

Children were recruited through early childhood educators (ECEs) at each ECEC and informed consent was obtained from the parents or legal guardians of all participating children. At the study onset, all children enrolled in each of the 8 participating ECECs were considered eligible to participate. Families were approached to participate in the PRO-ECO study by ECEs through an information and consent package. In addition, memebrs of the PRO-ECO research team were on-site at the ECECs during drop-off and pick-up times on selected days to support the completion of consent forms and answer questions about the PRO-ECO study. Consent forms could be completed electronically or on paper and ECE staff supported translation where necessary. Children were excluded from the study if parental consent was not received. Participating ECECs maintained continuous enrolment in the study from September 2021 to December 2022 so that new children entering their program were eligible to to participate during this time frame. When a new child enrolled in a participating ECEC, they received an information and consent package for participation in the PRO-ECO study. A total of 217 children aged 2.5 to 6 years attending a participating ECEC were included. Throughout the course of the study, an estimated 244 children were eligible to participate across the eight centres and three time points, indicating a recruitment rate of 88.93%.

**Table 2:** Characteristics of participating ECECs (*n* = 8).

Attribute	Intervention Group (Group 1)				Wait-list Control Group (Group 0)			
	Centre A	Centre C	Centre D	Centre G	Centre B	Centre E	Centre F	Centre H
Approximate size (m^2^)	335	754	196	173	171^a^	222	270	207
Grade^b^	Above-grade	At-grade	At-grade	At-grade	Above-grade	Above-grade	At-grade	At-grade
Surfacing materials	Concrete, natural soil/ dirt, mulch, artificial turf, wood decking^*^ (Fig. 2)	Concrete, natural soil/ dirt, mulch	Concrete, natural soil/ dirt, mulch, rocks	Concrete, mulch, natural soil/ dirt	Concrete, rubber, wood decking	Concrete, rubber, wood decking	Concrete, mulch, natural soil/ dirt	Concrete, mulch, natural soil/ dirt, gravel
Climbing structure	Play structure with slide, wood stumps	Climbing hill with slide, boulders, intertwined climbing logs^*^ (Fig. 3)	Play structure with slide, boulders, wood stumps	Play structure with slide, boulders	Play structure with slide	Play structure with slide	Play structure with slide, balance logs, wood stumps	Wood cubes, balance logs, wood stumps
Water feature	None	None	Rain catchers* (Fig. 4)	Water pump (moveable)* (Fig. 5)	None	None	None	Water pump & trough (fixed)
Sandbox	Yes	Yes	Yes	Yes	Yes	No	Yes	Yes
Mud kitchen	No	No	No	No	Yes	No	No	No
Table area	No	Yes	No	No	Yes	Yes	No	Yes

*These items were modified/added as part of the PRO-ECO intervention implementation (see Figs. 2, 3, 4 and 5).

^a^This centre increased in size minimally due to an extension to their outdoor space as part of the PRO-ECO intervention.

^b^Grade refers to the ground relationship to the building. Above grade indicates an outdoor play space above ground level. At-grade indicates an outdoor play space at ground level.

### Data collection

The primary quantitative outcome of the PRO-ECO study, children’s participation in play versus non-play behaviours while engaged in outdoor time at ECECs, was assessed using observational behaviour mapping (OBM), which collects information on children’s play behaviour in association with their outdoor environment^54,55^. Two measurement zones were created at each ECEC and on each data collection day, two researchers conducted independent observations alternating between the two zones approximately every 30 min^49^. Researchers scanned each zone in a counterclockwise direction and selected the first child to enter their viewpoint. Each play observation was video recorded and immediately coded on-site using ArcGIS Pro (version 2.9). Each video observation was 15-seconds in duration and was coded using the expanded version of the Tool for Observing Play Outdoors (TOPO) (Table 3)^56^. Factors with known associations with children’s play behaviour were also collected as part of the observational behaviour protocol, including gender, group size, adult interaction, play communication, physical activity intensity, risk-taking behaviour, peer interaction and environmental interaction. Gender was collected during observations and recorded based on how the child presented using potential visible gender markers, as outlined elsewhere by Loebach et al.^57^. Temperature and weather conditions were collected through the online website www.timeanddate.com/weather and matched to the day and time of data collection. Temperature was included as a continuous variable (°C), while weather conditions were further categorized into higher-level categories (cloudy, no rain; raining; sunny). Video observations of children were collected over the 3 data collection time points. Data collection at each time point was collected over approximately 4 days at each ECEC and during dedicated morning outdoor time as scheduled by the ECEC (9:30am – 12:00pm). The 3 data collection time points (October – November 2021; April – May 2022; and October – December 2022) sought to account for similar weather conditions across all data collection points. The research team completed a minimum of 200 observations per ECEC at each time point. A 10% sample of video observations were re-coded at each time point to examine the interrater reliability and agreement between coders. Conflicts were resolved within the team by reviewing the video observation and determining the final agreed-upon codes. The total number of collected observations is presented in the Results section.

To assess the primary outcome of the PRO-ECO study (play vs. non-play behaviour) we categorized play behaviour derived from the coded TOPO categories into a dichotomous play/non-play outcome variable. TOPO codes of non-play and restorative play were categorized as *Non-play* and physical play, exploratory play, imaginative play, play with rules, bio play and expressive play were categorized as *Play*. The categorization of all non-play and restorative play TOPO categories into *Non-play* for this analysis is based on the underlying viewpoint that play involves children actively engaged, whether self-directed or adult-directed, in a playful experience. Activities such as eating, self-care, and exclusively reading or resting are not considered as play behaviour within this study. As up to three TOPO codes could be assigned to each play observation, we determined additional rules to categorize play participation that were coded as non-play or restorative play and another play type (see Supplementary Material 2). Data on outdoor play participation were collected at the centre level to study children’s outdoor play across each participating ECEC, rather than studying individual children.

The reliability of data collection amongst coders was measured by the degree of interrater reliability and agreement, using weighted κ and intraclass correlation coefficients^58,59^. A κ value of 0.918 (agreement = 95.9%) was achieved prior to beginning data collection. Further information on the PRO-ECO wait-list control randomized control trial, including the data collection process, can be found in the previously published study protocol^49^.

**Table 3:** Tool for Observing Play Outdoors (expanded version), developed by Loebach and Cox^56^.

Play type and subtype	Description
Physical play	
Gross motor	Using large muscles, whole body movement, large muscle activities that require hand-eye coordination
Fine motor	Smaller muscle movements and hand-eye coordination, picking up or manipulating small objects
Vestibular	Activities that test and improve sense of balance or reinforce their relationship to the earth, movement of the head or quick movements in multiple directions
Rough and tumble	Engagement in playful or mock fighting or wrestling or more broadly playful physical contact
Exploratory play	
Sensory	Primarily passive (i.e., nonmanipulative) exploration of an object or environment, focused sensory attention
Active	Active manipulation of an object or the environment
Constructive	Physically building or constructing something or thoughtful destruction or taking apart of something
Imaginative play	
Symbolic	Using an object, action, or idea as a symbol for something else with no evidence of sociodramatic or fantasy
Sociodramatic	Pretending typical social, domestic, or interpersonal experiences or roles they may experience as adults
Fantasy	Enacting something that is unlikely to occur in real life
Play with rules	
Organic	2 or more kids agree to play or challenge each other in a certain way where they develop, negotiate, or change the rules as they go
Conventional	2 or more kids play games that have common, universal, or well-known rules that the players understand
Bio play	
Plants	Observes, discusses, or interacts with a living plant or parts of the plant (flowers or seed pods)
Wildlife	Observes, discusses, or interacts with wildlife (that is not a domestic pet)
Care	Acts in a way that demonstrates care or stewardship for the environment or an appreciation of nature
Expressive play	
Performance	Intentionally performing for others in some way
Artistic	Manipulating the environment specifically for an artistic, creative, or esthetic outcome
Language	Activities involving the playful use or testing of sound, words, or language
Conversation	Primary interaction is social conversation with children or adults
Restorative play	
Resting	Taking a mental break or rest
Retreat	Remove themselves to a small, controlled space, may watch others
Reading	Reading or writing for pleasure or listening to others or music
Onlooking	Child deliberately steps back from nearby play for a period of observation
Non-play	
Self-care	Taking care of themselves or their appearance, can include helping another with these activities
Nutrition	When a child is taking a break to eat or drink
Distress	When a child is disengaged from play and exhibiting signs of distress
Aggression	Refers to non-playful, antagonistic interactions with another child or adult
Transition	Non-playful movement from one space to another, no active engagement or exploration of the environment
Other	Other types of observed “non-play” activities, can include “chores” or cleanup work

### Analysis

**Fig. 6:**
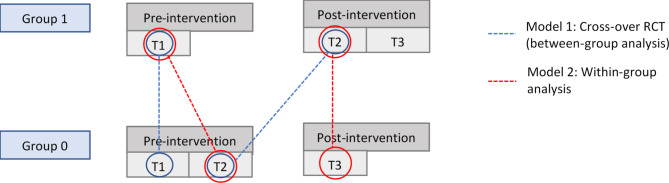
Between-group and within-group PRO-ECO analyses data diagram.

The proportion of play participation in comparison with non-play participation across ECECs at each time point were summarized by intervention group using frequency and percentages. Intervention effect was assessed using a cross-over randomized control trial (RCT) study design. The primary analysis used a mixed effect logistic regression model to investigate the differences in the primary dependent outcome, *play participation* (play vs. non-play), between the intervention and wait-list control groups (Model 1), as well as within-group comparisons (Model 2) (Fig. 6). We used random effects in mixed effect models to account for observation clustering within the same care centers. In all models, we controlled for known covariates, identified a priori, that could confound the associations: *weather conditions* (sunny, cloudy and no rain, and raining); *temperature* (Celsius degrees, as a continuous variable); and *gender* (boy and girl).

For the between-group comparison, the independent variables included in the model were *group* (Group 0: waitlist-control; and Group 1: intervention group), *absolute time* (Time 1 and Time 2), and the *absolute time by group* interaction. For within-group comparisons, the independent variable included in the model was *relative time* (pre- and post-intervention). In the within-group comparisons, we also included *group* and *relative time by group* interaction to explore if time trends differed by group; with any significant relative time by group interaction, we explored pre- and post- time trends separately by each group (Model 3 and Model 4). All analyses used all available (complete-case) data. All statistical analyses were performed using R-4.2.2. Mixed effect models utilized the “lme4” R package.

## Results

### Descriptive findings

Table 4 provides the detailed data collection efforts across the eight centers. Across all time points, a total of 5,213 observations were collected as part of the PRO-ECO study. These observations were collected over 337 on-site hours at the eight participating ECECs and resulted in 1,303 min of observational data. While the research team aimed to collect approximately 200 observations at each centre for each data collection time point, the total number of observations included in the study sample ranged from 191 to 253, per centre and data collection time point, following the data cleaning process. At each data collection time point, similar total numbers of observations were collected with 1,726 observations collected at Time 1 (baseline), 1,761 observations collected at Time 2 (6-month follow-up) and 1,726 observations collected at Time 3 (12-month follow-up). Table 4 also provides an overview of the time spent by the research team on-site collecting observations, the number of collected observations and the total minutes of observation data collected, by participating centre and data collection time point.

Across all observations (*n* = 5,213), 80.7% were play behaviour observations, and 19.3% were non-play behaviour observations. Among the wait-list control group (Group 0), the proportion of play participation at each time point was: 78.6% for Time 1; 79.2% for Time 2; and 76.8% for Time 3. Among the intervention group (Group 1), the proportion of play participation at each time point was: 83.7% for Time 1; 86.0% for Time 2; and 79.9% for Time 3. Six months following implementation of the PRO-ECO intervention, the wait-list control group saw a decrease in the proportion of play participation (from 79.2 to 76.8%), whereas the proportion of play participation stayed relatively stable for the intervention group (from 83.7 to 86.0%). Temperature varied slightly across each data collection time point: Time 1 temperatures were between 7 °C and 14 °C (Mean: 10 °C); Time 2 temperatures were between 3 °C to 15 °C (Mean: 9 °C); and Time 3 temperatures were between − 2 °C to 15 °C (Mean: 7 °C). Table 5 further provides an overview of study measures across intervention groups at each time point.

**Table 4:** Hours On-site collecting observations, number of collected observations and Time (minutes) of Observation Data, by Centre and Data Collection Time Point.

Centre	Time 1			Time 2			Time 3			Total		
	Hours On-site Collecting Observations	Number of Observations (*n*)	Minutes of Observation Data (min)	Hours On-site Collecting Observations (hrs)	Number of Observations (*n*)	Minutes of Observation Data	Hours On-site Collecting Observations (hrs)	Number of Observations (*n*)	Minutes of Observation Data	Hours On-site Collecting Observations (hrs)	Number of Observations (*n*)	Minutes of Observation Data
Centre A	14.00	215	53.75	12.60	205	51.25	11.30	221	55.25	37.90	641	160.25
Centre B	14.30	219	54.75	13.40	220	55.00	11.80	210	52.50	39.50	649	162.25
Centre C	16.90	226	56.50	13.10	209	52.25	15.10	215	53.75	45.10	650	162.50
Centre D	20.30	217	54.25	12.10	214	53.50	12.80	203	50.75	45.20	634	158.50
Centre E	14.20	224	56.00	18.40	221	55.25	12.90	207	51.75	45.50	652	163.00
Centre F	12.17	205	51.25	15.90	217	54.25	14.80	210	52.50	42.87	632	158.00
Centre G	14.50	191	47.75	13.10	222	55.50	13.40	238	59.50	41.00	651	162.75
Centre H	14.30	229	57.25	15.40	253	63.25	10.70	222	55.50	40.40	704	176.00
Total	120.67	1,726	431.50	114.00	1,761	440.25	102.80	1,726	431.50	337.47	**5**,**213**	**1**,**303.25**

**Table 5:** Descriptive sample characteristics of all observations (model 1) across ECECs in the PROmoting early childhood outside (PRO-ECO) study, stratified by intervention arm.

Measures		Time 1	Time 2	Time 3
Group 0		Pre-intervention		Post-intervention
# Observations (N)		877	911	849
Play Participation (%)		78.6	79.2	76.8
Weather Conditions (%)	Sunny	18.8	24.7	24.6
	Cloudy, no rain	57.3	58.3	61.4
	Raining	23.8	17.0	14.0
Gender (%)	Boy	60.9	56.9	59.6
	Girl	39.1	43.1	40.4
Temperature °C (Mean, SD)		9.7 (1.7)	8.1 (2.0)	6.9 (4.3)
Group 1		Pre-intervention	Post-intervention	
# Observations (N)		849	850	877
Play Participation (%)		83.7	86.0	79.9
Weather Conditions (%)	Sunny	24.0	11.1	27.9
	Cloudy, no rain	62.2	60.1	72.1
	Raining	13.7	28.8	0.0
Gender (%)	Boy	54.5	61.2	58.4
	Girl	45.5	38.7	41.6
Temperature °°C (Mean, SD)		10.6 (2.0)	10.9 (1.6)	7.7 (3.9)

### Between-group comparisons of proportion of play participation in absolute time

**Fig. 7:**
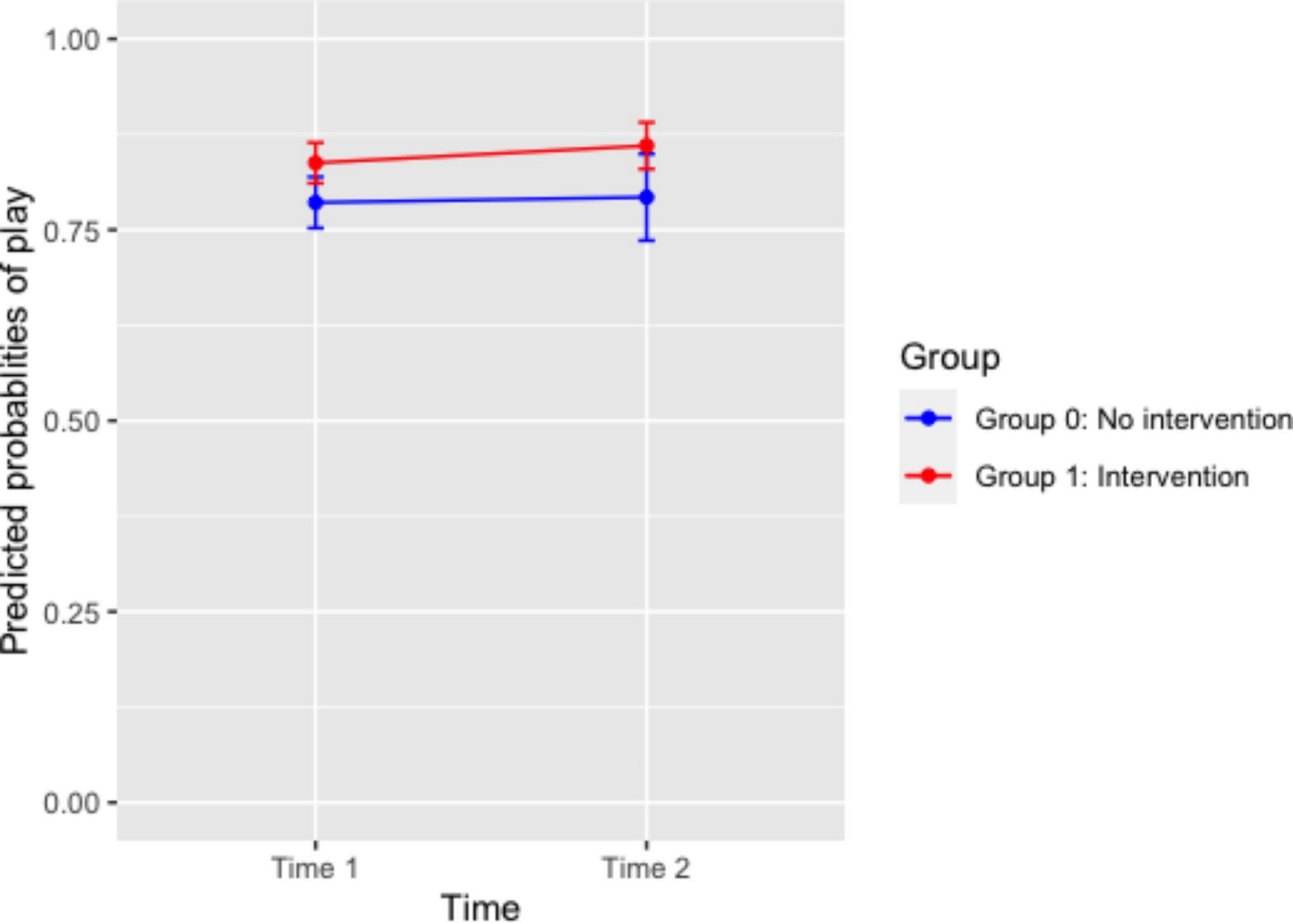
Predicted probabilities of play between Group 0 and Group 1 at Time 1 and Time 2, with 95% CI.

The primary logistic regression analysis (Model 1) showed no significant effect of absolute time, group, nor absolute time by group interaction (Table 6). Figure 7 plotted the predicted probabilities of play participation for Group 0 and Group 1, when all covariates are the same and indicates that there was no significant change in play participation between pre-PRO-ECO intervention and post-PRO-ECO intervention, between the two groups. Among other variables, only weather condition was significantly associated with play participation: in comparison to sunny weather conditions, rainy weather conditions were negatively associated with play participation (OR = 0.71, 95% CI = 0.53, 0.94). A higher temperature also corresponds with higher odds of play (OR = 1.05, 95% CI = 1.00, 1.11). Gender did not show a significant effect on play participation.

**Table 6:** Mixed-effect logistic regression results (odds ratios, 95% CI) examining the intervention effect on play participation in absolute time.

Variables	Model 1: Odds Ratios (95% CI)		
Absolute Time^a^	1.15	(0.89, 1.47)	
Group	1.24	(0.66, 2.33)	
Absolute Time X Group	1.06	(0.73, 1.55)	
Weather Conditions (%)			
Sunny	Reference		
Cloudy, no rain	0.99	(0.78, 1.25)	
Raining	0.73	(0.55, 0.96)	*
Gender (%)			
Boy	Reference		
Girl	0.94	(0.79, 1.13)	
Temperature °C (Mean, SD)	1.05	(1.00, 1.11)	*

Note: significance level *p < 0.05, **p < 0.01, ***p < 0.001.

^a^Absolute Time: real time of data collection time points.

### Within-group comparisons of proportion of play participation in relative time

**Fig. 8:**
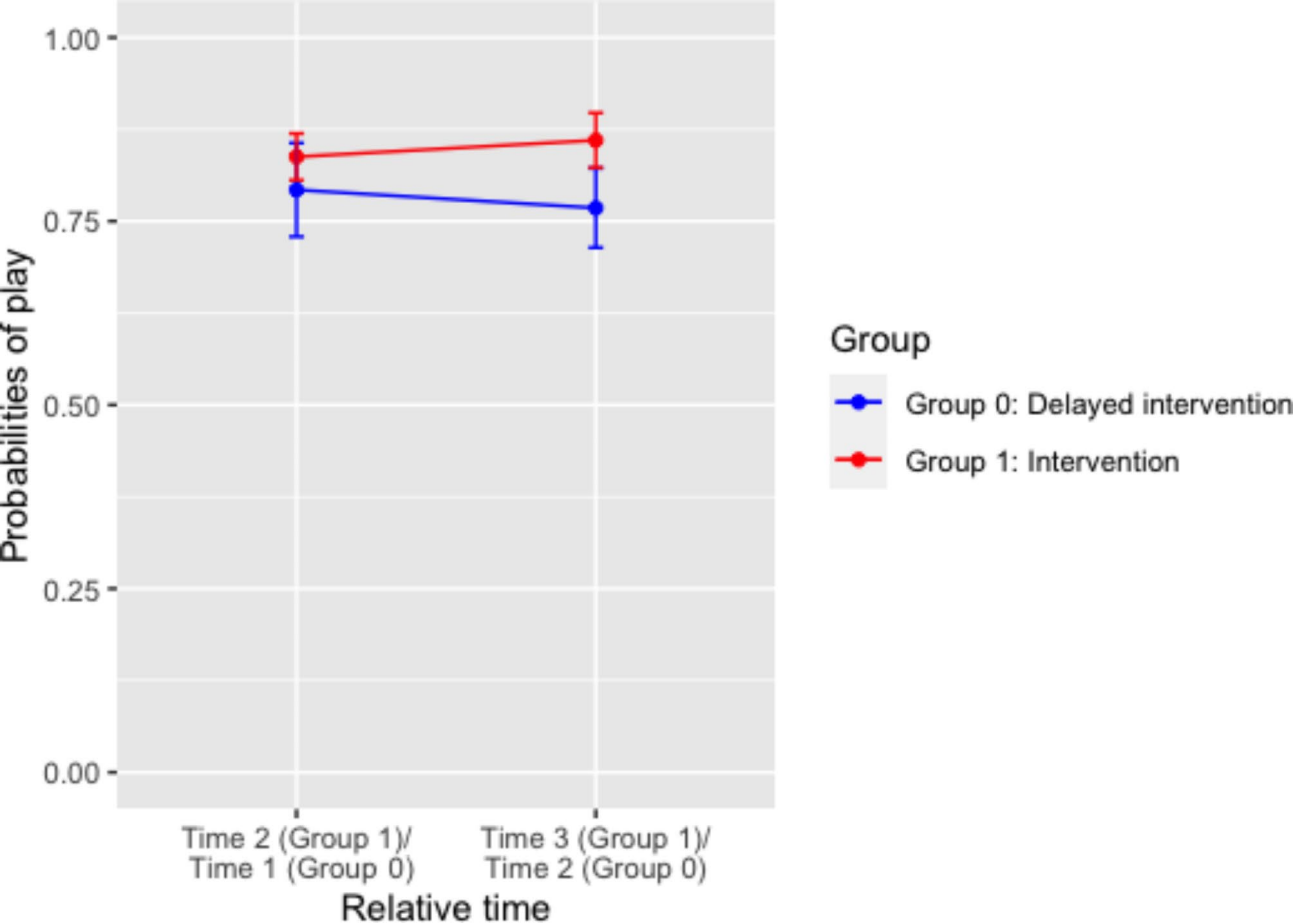
Predicted probabilities of play between Group 0 and Group 1, pre- and post-intervention, with 95% confidence interval.

In Table 7, the logistic regression analysis (Model 2) shows no significant effect of relative time or group, but a significant relative time by group interaction (OR = 1.51, 95% CI = 1.05, 2.16). Figure 8 plotted the different time trends of play participation between Group 0 and Group 1, pre- and post-intervention. Among other variables, weather condition was significantly associated with play participation: in comparison to sunny weather conditions, rainy weather conditions were associated with lower odds of play participation (OR = 0.53, 95% CI = 0.40, 0.71). Other variables, including relative time, gender and temperature, did not show significant effects on play participation. Given a significant relative time by group interaction, time trends of play participation were examined within each group (Model 3 and Model 4). Table 8 shows changes in play participation for each group pre- and post-intervention. For Group 0, there was no significant change in play participation pre- and post- intervention. For Group 1, there was no significant change, however the estimate was positive (OR = 1.28, 95% CI = 0.97, 1.70) in the change in play participation from Time 1 to Time 2. There was no significant change in play participation in Time 3.

**Table 7:** Mixed-effect logistic regression results (odds ratios, 95% CI) examining the time trends in relative time on play participation.

Variables		Model 2: Odds Ratios (95% CI)		
Relative time^a^		0.86	(0.68, 1.08)	
Group		1.24	(0.83, 1.84)	
Relative Time X Group		1.51	(1.05, 2.16)	*
Weather conditions (%)	Sunny	Reference		
	Cloudy, no rain	0.84	(0.66, 1.06)	
	Raining	0.53	(0.40, 0.71)	***
Gender (%)	Boy	Reference		
	Girl	0.92	(0.77, 1.10)	
Temperature °C (Mean, SD)		1.02	(0.99, 1.06)	

Note: significance level *: *p* < 0.05, **: *p* < 0.01, ***: *p* < 0.001.

^a^ Relative Time: pre- vs. post-intervention.

**Table 8:** Mixed-effect logistic regression results (odds ratios, 95% CI) examining the time trends in relative time by group on play participation.

		Model 3: Odds Ratios (95% CI) Group 0	Model 4: Odds Ratios (95% CI) Group 1	
		Time 3 vs. Time 1	Time 2 vs. Time 1	Time 3 vs. Time 1
Relative time^a^		0.85 (0.67, 1.07)	1.28 (0.97, 1.70)	0.92 (0.70, 1.23)
Weather Conditions (%)	Sunny	Reference	Reference	Reference
	Cloudy, no rain	0.81 (0.59, 1.10)	0.91 (0.61, 1.34)	0.98 (0.74, 1.30)
	Raining	0.50 (0.35, 0.72) ***	0.60 (0.38, 0.93) *	1.28 (0.70, 2.37)
Gender (%)	Boy	Reference	Reference	Reference
	Girl	0.81 (0.64, 1.02)	1.09 (0.83, 1.43)	0.90 (0.70, 1.15)
Temperature °C (Mean, SD)		1.02 (0.98, 1.06)	1.04 (0.97, 1.13)	1.05 (1.01, 1.09) *

Note: significance level * < 0.05, **p < 0.01, ***p < 0.001.

^a^ Relative Time: pre- vs. post-intervention.

### Centre comparisons of proportion of play participation

**Fig. 9:**
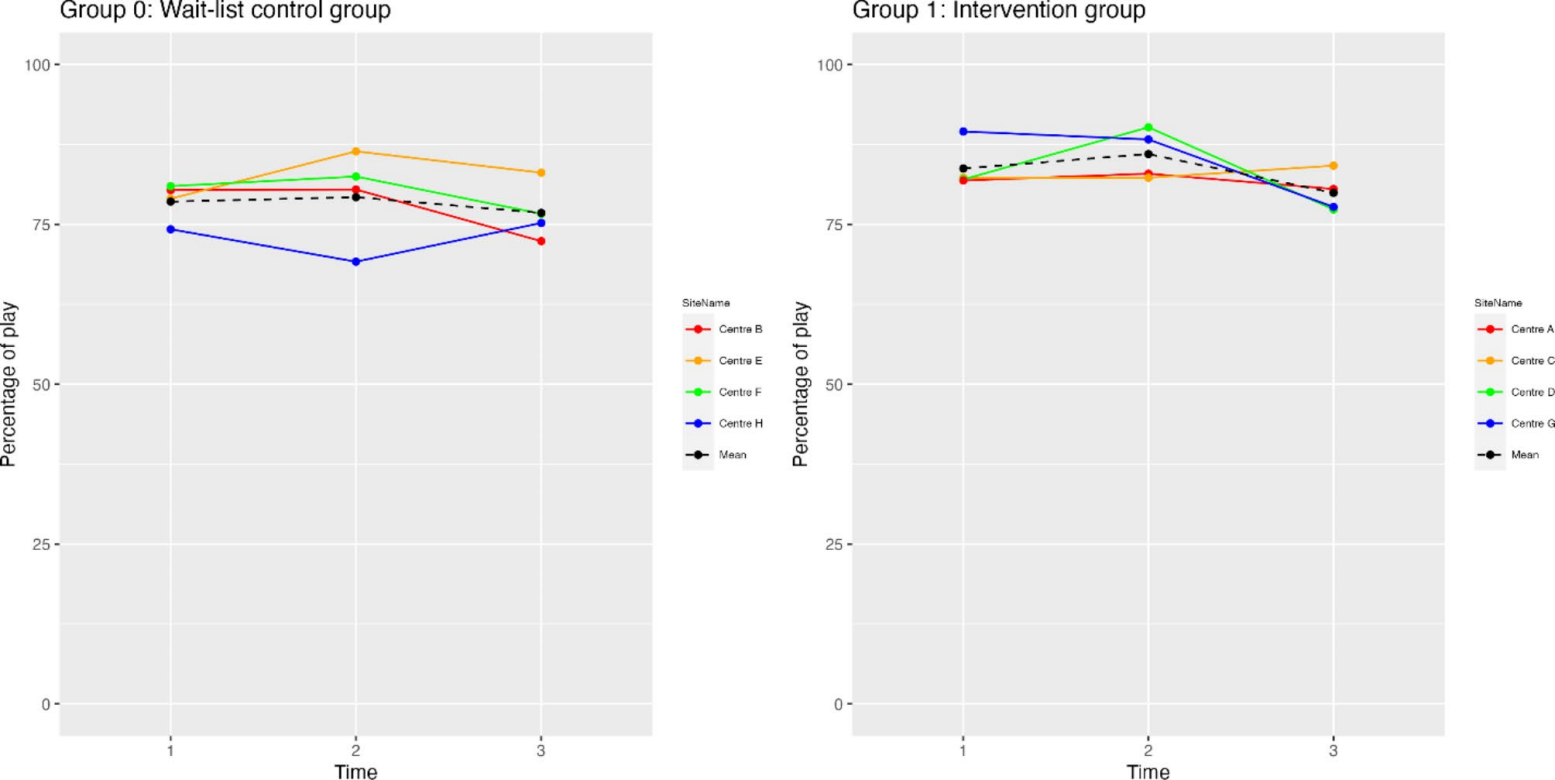
Centre-specific play participation rates, by group and data collection time point.

To understand the change in play participation rate for each of the ECECs across the data collection time points, the percentage of play by intervention group was graphed by participating centre at each time point of data collection (Fig. 9). It is evident that play participation varied widely across ECECs and can help to explain the null finding for Model 1. For example, the trends in play participation rates were not always linear following implementation of the PRO-ECO intervention, nor were they stagnant between data collection time points where no intervention occurred. When comparing trends in play participation rates by ECEC, some centres performed as hypothesized, with increases in play following the PRO-ECO intervention, whereas other centres saw no change or even decreases.

Among ECECs in Group 1, Centre D experienced a large increase in play participation in the short-term following the intervention implementation (between Time 1 and 2), but a large drop-off between Times 2 and 3. In contrast, Centre A, C and G had relatively unchanged play participation rates following the PRO-ECO intervention at Time 2. Centre C showed a modest increase at Time 3, indicating that there may have been delayed effects of the intervention that did not appear immediately following its implementation at Time 2. Both Centre D and Centre G had declines in children’s play participation rate from Time 2 to Time 3, indicating that any immediate effect of the PRO-ECO intervention may have worn off over time. Among ECECs in Group 0, Centre H was the only ECEC that experienced an increase in play participation following implementation of the PRO-ECO intervention (between Time 2 and 3) (Fig. 9). Despite not having received the PRO-ECO intervention at Time 2, two centres (Centre E and F) had increases in their play participation rate between Time 1 and Time 2. However, both of these centres experienced drops in play participation between Times 2 and 3 after receiving the PRO-ECO intervention. Centre E did continue to see a higher play participation rate at Time 3 than at Time 1, indicating that there was an increase in play participation throughout the duration of the study. While retaining a relatively constant play participation rate between Time 1 and Time 2, Centre B saw a sharp decline in play participation at Time 3 following implementation of the PRO-ECO intervention.

### Frequency of play behaviour types

The TOPO play typology framework supported the coding of eight different types of play behaviour. Table 9 outlines the proportions of different play behaviour types observed within each intervention group pre- and post-intervention. Future analyses will explore the change in diversity of play, specifically how the PRO-ECO intervention may have influenced increases or decreases in specific play types, as well contributed to the overall diversity of children’s play behaviour.

**Table 9:** Proportion of play behaviour type, by intervention group and time point.

Measures		
Group 0	Pre-intervention (Time 1 and Time 2)	Post-intervention (Time 3)
# Observations (N)	1,788	849
Play Behaviour Type [n(%)]		
Physical Play	1102 (61.6%)	512 (60.3%)
Exploratory Play	573 (32.0%)	242 (28.5%)
Imaginative Play	130 (7.3%)	53 (6.2%)
Play with Rules	76 (4.3%)	21 (2.5%)
Bio Play	75 (4.2%)	28 (3.3%)
Expressive Play	307 (17.2%)	165 (19.4%)
Restorative Play	294 (16.4%)	116 (13.7%)
Non-Play	258 (14.4%)	139 (16.4%)
Group 1	Pre-intervention (Time 1)	Post-intervention (Time 2 and Time 3)
# Observations (N)	849	1,727
Play Behaviour Type [n(%)]		
Physical Play	501 (59.0%)	1,102 (63.8%)
Exploratory Play	361 (42.5%)	672 (38.9%)
Imaginative Play	75 (8.8%)	146 (8.5%)
Play with Rules	34 (4.0%)	54 (3.1%)
Bio Play	47 (5.5%)	73 (4.2%)
Expressive Play	148 (17.4%)	271 (15.7%)
Restorative Play	123 (14.5%)	246 (14.2%)
Non-Play	97 (11.4%)	163 (9.4%)

## Discussion

The PRO-ECO intervention included four key components that address common barriers to children’s outdoor play, as supported by existing literature^60–64^. This study was not designed to analyze the effect of each intervention component separately on children’s outdoor play participation, but rather the influence of these components collectively. Previous research has focused on interventions that increase specific types of play, such as active or physical play, rather than a comprehensive view that includes the many forms of children’s outdoor play^65–67^. To add to the literature, this study considered a holistic definition of outdoor play, encompassing many different forms of play, including physical, exploratory, imaginative, bio, play with rules, and expressive. The results of this pilot randomized control trial revealed no significant change in the proportion of play participation following implementation of the PRO-ECO intervention. We hypothesize two distinct reasons for these findings: (i) high rates of play participation prior to the intervention implementation leading to a ceiling effect; and (ii) challenges experienced in the implemention and maintenance of the PRO-ECO intervention.

### High-levels of baseline play participation at ECECs

This study revealed high proportions of children’s play participation, in comparison to non-play participation, at pre-PRO-ECO intervention data collection time points which may have contributed to a ceiling effect within our data^68^. The percent of play participation prior to implementation of the PRO-ECO intervention was 78.9% within the wait-list control group (Group 0) and 83.7% within the intervention group (Group 1).The high proportions of play participation prior to any intervention indicates that there may have been limited opportunity for improvement in overall play participation. To our knowledge, there are limited other studies that have measured play vs. non-play in ECEC settings, therefore it is challenging to understand if the high levels of play exhibited within our study are common across the literature. A recent study by Storli et al.^69^ reported that 30.9% of their outdoor video observations in ECECs were non-play behaviours. Additional studies^70,71^ have measured play and non-play behaviours among older children in school settings and found observed play participation rates between 40.9% and 53.0% at baseline. However, it is important to note that the definition of non-play is not universal and is determined by the methodology implemented to categorize children’s behaviours. The methodology for categorizing children’s non-play behaviour (Supplementary Material 2) was unique to our study.

The observed activities and behaviours classified as non-play (e.g. eating, distress, aggression and self-care) had low incidences across our observations. Due to the scheduled nature of outdoor time across participating ECECs, non-play behaviours may not occur as frequently due to the limited amount of time that children have to spend outdoors. Children might have preferred to participate in play behaviours instead of non-play behaviours, such as eating or self-care breaks, because of the short duration of outdoor time they had available. In addition, some non-play behaviours were expected to remain stable in ECEC settings even after implementation of the PRO-ECO intervention, such as scheduled lunch times which would occur regardless of changes to a ECEC program or space. Overall, the high proportion of play participation at pre-intervention time points indicates that additional outcome measures, beyond a dichotomous play participation variable, are required to evaluate comprehensive outdoor play interventions that support children’s outdoor play participation in ECECs.

### PRO-ECO intervention implementation and sustainability challenges

Overall, children’s outdoor play participation, versus non-play participation, did not consistently increase as a result of the PRO-ECO intervention, though the effect size was positive (Table 6). While there was no significant increase in play participation between or within the intervention groups, two centres (1 in Group 1; 1 in Group 0) experienced an increase in children’s outdoor play participation following implementation of the PRO-ECO intervention. Two additional centres in Group 1 (Centre A and C) experienced minimal change in children’s play participation between Time 1 and Time 2. This finding highlights the variability of the intervention in implementation and uptake across ECECs, and that there are important considerations to the planning, implementation and sustainability of outdoor play interventions.

All four components of the PRO-ECO intervention experienced limitations to implementation and sustainability. The primary challenges that impacted the PRO-ECO intervention implementation included the identification of outdoor play values and the implementation of new policy, the engagement of families during the COVID-19 pandemic, and the delivery of ECE professional development and training. All participating ECECs experienced challenges to sustaining the PRO-ECO intervention over the study timeline, as well as beyond the study completion date. In particular, sustaining the uptake of the ECE training and maintaining the built environment modifications was constrained due primarily to ECE turnover, as well as seasonal changes in the physical environment.

#### Policy changes and implementation

As a central component to the PRO-ECO intervention, the identification of outdoor play values and their policy implications was intiatied by the YMCA GV management team, who developed the values document and disseminated it to the ECECs. The ECECs were then responsible for applying it to ECEC-level policies and procedures. However, this process was delayed due to challenges in managing the COVID-19 pandemic, such that it was not distributed to Group 1, the intervention group ECECs, until we began Time 2 data collection, giving insufficient time for implementation. Further, the top-down approach to values identification (rather than having each ECEC identify their own values) may have contributed to limited uptake of the values and policy change. Despite these challenges and delays, postiive changes were implemented by the YMCA GV in earlier stages that stemmed from discussions to identify their outdoor play values. For example, encouraging ECEs to implement practices that made it easier for children who wanted to remain outside longer than the time their group had been allotted to do so. The family engagement component of the PRO-ECO intervention also experienced limitations due to COVID-19 restrictions that were present throughout the study timeline. Public health and centre-specific restrictions were in place at the start of the study and curtailed parents’ access to the ECECs, influencing the extent and type of parent engagement opportunities available. Following easing of restrticitons, many families continued to follow their pandemic-related norms and limited their presence within the physical ECE space. These parameters made in-person events limited or impossible in many of the participating ECECs.

#### ECE training

The implementation of the ECE training underwent numerous challenges. In addition, there were challenges related to the sustainability of the training throughout the course of the PRO-ECO intervention, as well as post-project. The length and style of educator training differed between the two intervention groups partly due to COVID-related constraints. Group 1 received an in-person full day training, whereas Group 0 received a half-day online training. The training provided as part of the PRO-ECO intervention was developed and provided by the YMCA of Southwestern Ontario and was in the process of being expanded to include YMCA ECECs across Canada. The ECECs that were part of PRO-ECO were among the first to receive it as part of this expansion. As can be typical with rapid expansions, early adoption reveals ‘growing pains’ that can be addressed in subsequent iterations. Evidence from the first ECECs trained in Ontario, prior to expansion of the training across Canada, demonstrated that the training improved educator self-efficacy but not knowledge or risk tolerance^72^. This finding indicates that a more comprehensive and extensive training program is required to create significant and lasting educator behavioural change within ECEC settings.

Another serious challenge to uptake of educator training was staff turnover and attrition, which previous research indicates contributes to the quality of professional practice and pedagogical leadership^73^. This study experienced first-hand the ECE shortage among the workforce that was exacerbated by the COVID-19 pandemic, resulting in challenges with retention of ECEs, and consequently the uptake of the ECE training associated with the PRO-ECO intervention. The effect of the PRO-ECO intervention on children’s outdoor play participation may have waned in conjunction with the reduced impact of the training over time. To address staff turnover, a ‘booster’ training session was provided to all new staff prior to the Time 3 data collection. However, staff turnover was so persistent that even this additional training provision may not have been sufficient. An additional training-related challenge experienced in this project was the limited on-going mentorship opportunities for ECEs. The training was designed by the YMCA of Southwestern Ontario to be supplemented by regular check-ins (such as at monthly staff meetings) to raise and address emerging issues and provide mentorship support. These sessions were largely absent due to reduced capacity and staff turnover. Overall, the availability of more frequent opportunities for training of new ECEs, as well as robust ongoing mentorship support may have improved the impact of the training and outcomes for the study, as also identified by previous studies^74,75^.

#### ECEC outdoor space modification

Changes to the participating ECECs built environment as part of the PRO-ECO intervention aimed to enhance available outdoor affordances, loose parts, natural materials and opportunities for challenge. The implementation of this component was performed in partnership with the UBC School of Architecture and Landscape Architecture, the YMCA GV and each ECEC, and the relevant licensing officer. Previous research has identified important considerations for maintaining sustainable outdoor environment modifications, including implementing low maintenance, native plants compatible to the region^76^, providing self-sufficient irrigation systems^76^, supporting gardening groups^76^, and including the cost of outdoor maintenance within the intervention parameters^77^. In addition, when practitioners are included in the development of the outdoor play space built environment modifications, a stronger ownership of the space is established, leading to more care for maintaining the space^78^.

The implementation of the PRO-ECO intervention experienced climate change related extreme weather conditions, including atmospheric rivers, hail and snow, and drought, impacting planting. Many of the environment modifications required routine upkeep, such as maintaining planting areas and storing new loose parts and equipment in undercover storage, of which the responsibility fell with each individual centre and was difficult to monitor. While the PRO-ECO intervention strived to follow best practice for built environment modification, the maintenance of the modifications required follow-up from the ECECs that was difficult to sustain and may have contributed to the reduced impact of the PRO-ECO intervention on children’s outdoor play over time. Previous studies have outlined similar challenges, including barriers to storing loose parts within the outdoor play space^79,80^ and maintaining aspects of the outdoor play environment^81^. Many natural materials, such as those implemented as part of the PRO-ECO intervention, required continuous replenishment (such as mulch, soil and loose parts) to ensure consistent availability. In addition, the seasonal nature and time required for plantings to fully develop may have not aligned with this study’s scheduled post-intervention data collection time points. Post-intervention data collection in the summer season or at a time period longer then 12-months post-intervention may have illuminated different play behaviour changes among participating children. In addition, further collaboration with the ECEs at each participating ECEC when designing and implementing these components of the PRO-ECO intervention may have supported the sustainability and maintenance of the built environment modifications long-term.

#### Additional considerations for the development and implementation of an outdoor play intervention at ECECs

The results of this study show that temperature and weather played a significant role in children’s outdoor play participation. Specifically, higher temperatures increased children’s outdoor play participation, while rainy weather decreased play participation, similar to other studies that have found children’s outdoor physical activity and play increases in higher temperatures (between 0 and 20 °C) and weather conditions where there is no precipitation^82–84^. While this study aimed to collect data at similar seasonal time points (Fall/Spring), the temperature range varied at each data collection time point. Time 3 data collection experienced cooler temperatures (below 0 ^o^C and light snow) which was not present in Time 1 or Time 2 data collection. The variance in temperature conditions was also seen between the intervention groups, where the wait-list control centres (Group 0) experienced a higher proportion of rainy weather conditions and lower mean temperature than the intervention centres (Group 1). While our analysis controlled for weather and temperature, the impact of the variance in weather conditions across data collection time points and between intervention groups on the ECEC space provision and the ECE practices are less known. In addition, the significant role that weather and temperature have on children’s play participation may influence the success of outdoor play interventions and should be considered within future intervention planning.

Despite the implementation of a comprehensive, evidence-informed outdoor play intervention, the results of this study indicate that barriers continue to exist towards enhancing children’s outdoor play behaviour at ECECs. In particular, there were large differences in outdoor play participation at baseline and following implementation of the PRO-ECO intervention between each participating ECEC. The efficacy and effect of the PRO-ECO intervention appears to be specific to each ECEC, rather than consistently received across all participating centres. However, it is important to consider that each PRO-ECO intervention was tailored to the individual ECEC, resulting in non-uniform aspects of the intervention across the project. For example, Centre C (Group 1) saw increases in children’s play, versus non-play, participation following implementation of the PRO-ECO intervention and this change continued to be present at 12-month follow-up. This participating centre received one of the most innovative built environment modifications with the construction of a unique natural climber in an underutilized open space (see Figs. 3 and^50^ for more information). Centre C was the largest participating centre and had the necessary ground surface in place to facilitate this built environment change. The ability to implement a comprehensive structure in this space may have contributed to more sustainable outdoor play behaviour change within children at this centre. In comparison, Centre B had the smallest physical outdoor space that was already largely occupied by fixed structures prior to implementation of the PRO-ECO intervention. This reduced the project team’s ability to implement unique built environment features, beyond planting and loose parts. A component of Centre B’s built environment modification focused on removing an existing fixed structure surrounding the sandbox (see Supplementary Material 1 Figs S3-S4 and^50^ for more information) that inhibited children from navigating the play space. The proportion of play participation, in comparison to non-play participation, decreased following implementation of the PRO-ECO intervention, which may be as a result of the limited built environment modifications available within the existing constrained outdoor play space at Centre B. Further analyses within this study will seek to understand and describe the centre-level differences that may have contributed to the diversity of change in children’s play specific to the ECEC-level.

### Strengths and limitations

To our knowledge, this is the first cluster randomized controlled trial to evaluate a comprehensive outdoor play intervention in ECECs. Among the strengths of this study was the interdisciplinary stakeholder committee that was gathered to inform best practices and the primary components of this intervention. The development of the PRO-ECO study and the PRO-ECO intervention included extensive partnership and consultation with each participating ECEC, ECEs, licensing officers, and multidisciplinary experts in early childhood education, landscape architecture, public health, outdoor play, psychology and child development. The research team considered the geographic area and socio-economic characteristics of the community and performed stratified randomization to allocate participating ECECs into the intervention and control arms of the study. This process supported a diverse and representative sample of ECECs, however, we could not account or stratify for all ECEC characteristics.

The YMCA is a large national organization with many ECECs throughout the country and the region. This was a strength of this study in that it could draw on resources that may not be available to smaller organizations or stand-alone ECECs. In addition, partnership with a large national organization offeres opportunities for expansion of practices, like the PRO-ECO intervention, across all of their ECECs. It can also represent a limitation, which was evident in the implementation of the Outdoor Play Policy component of the PRO-ECO intervention, which followed a top-down approach and might have limited a sense of understanding and ownership at each ECEC for the sentiments contained within the values identified by the YMCA GV management team.

Due to the complexity of this study design, there are limitations that are present within this study. First, this is a pilot study and the sample size was based on feasibility while retaining optimal statistical power. A larger sample may have facilitated identification of the effect size between the intervention and wait-list control groups, particularly as play participation was high in all ELCCs at baseline. An additional limitation of this study was the occurrence of the COVID-19 pandemic that impacted the operations of participating ECECs. Onboarding of ECECs and consenting children occurred in mid-to-late 2021, when many COVID-19 restrictions were in place in BC, thus limiting the research team’s capability to support ECECs in the consenting and data collection practices. In addition, ECECs experienced unusually high staff-turnover during this time and outdoor play practices may have been impacted. To account for the possible limitations due to COVID-19 restrictions, the research team connected with participating ECECs frequently and made field notes to address potential changes in normal routines.

A limitation to this study’s analysis included the dichotomization of children’s play behaviour into a binary play or non-play outcome variable. A dichotomous primary outcome variable was analyzed to assess the impact of the PRO-ECO intervention on children’s play participation, in comparison to non-play participation which included activities such as eating, self-care, distress or aggression. However, this approach masks the heterogeneous nature of chldren’s play and contributed to the ceiling effect apparent in the high proportions of play participation noted at pre-intervention time points. Further, this variable was derived through an extensive process to systematically categorize each observation into either a play observation or non-play observation which may be subject to error (see Supplementary Material 2). Further analyses from this study will seek to understand changes in children’s diversity of play and strive to consider a holistic view of children’s play behaviour that considers the many forms of play that can occur within a given observation.

In conceptualizing and designing this study, data were collected at three time points (baseline, 6-month follow-up and 12-month follow-up) to assess short- and longer-term outcomes of the PRO-ECO intervention. However, the seasonal timing and shorter follow-up time of the data collection time points may have contributed to a limitation of our data collection process. Future studies should consider increasing the number of data collection time points to account for all seasons and collecting data over a longer time period to assess multiple short-term and long-term time points. More frequent data collection may support a more robust understanding of outdoor play behaviour change, while being able to adequately control for the diverse weather and other confounding environmental influences. Lastly, the two-year data collection phase of the PRO-ECO study exhibited a high variability of weather, temperature and precipitation patterns, including heat waves, atmospheric rivers and intense hail, consistent with the increasing effects of climate change. While we controlled for weather conditions and temperature within our analysis, we expect that the extreme weather patterns seen in this study may have contributed to additional effects beyond what can be controlled in a statistical model. Future studies should consider collecting data simultaneaously across participating ECECs to ensure that weather patterns are comparable on data collection days for each centre. The generalizability of our findings are most applicable to geographic areas with similar weather patterns of the Greater Vancouver region.

### Implications

The primary findings from the PRO-ECO randomized control trial demonstrate that future considerations for comprehensive outdoor play interventions are required to successfully influence children’s outdoor play behaviour at ECECs. Consideration of the sustainability and scalability of an intervention project is imperative to ensure long-term and viable change that encompasses human behaviour, such as ongoing training and mentorship, and support for landscape maintenance. In addition, building connections with educators, families and children is essential to an ECEC’s sustainable support of outdoor play. Importantly, further analyses will focus on how the PRO-ECO intervention influenced additional child outcomes, including the diversity of play. Future studies should be wary of limiting their primary or solitary outcome measure to a binary play vs. non-play variable.

There is an opportunity for the PRO-ECO intervention to connect with Indigenous Ways of Knowing and Being, which emphasize connection to and learning from the Land. Encouraging children’s time in the outdoors and learning about the land that they are on, including about the local Indigenous communities, is an important part of supporting reconciliation. Collaboration with local Indigenous communities could help weave these principles into each aspect of the intervention to promote children’s and ECEs’ stewardship and care for and connection to the land, such as in the selection of plants, learning about their traditional uses, importance or symbolism. Métis herbalist and educator, Lori Snyder conducted a workshop with the SALA students, sharing her extensive knowledge of and practice with native plants, guiding their selections and donating plant material for the project^50^. While a promising first step, future work should partner with Indigenous communities from the outset and weave these principles throughout the project.

Comprehensive outdoor play interventions implemented within ECEC settings can showcase results at the educator-level in addition to the child-level. Observationally, we saw that professional development training with educators supported enhanced excitement for the outdoors and willingness to play outside longer in all weather, which in turn, increased children’s willingness to be outdoors. The PRO-ECO project sparked innovation and ECEs began creating new opportunities to encourage child-directed choices outside. At one participating centre, following the PRO-ECO intervention, educators supported children to ride down a steep slope on their tricycles – an activity that was previously seen as too risky and educators were hesitant to support it.

## Conclusion

The PRO-ECO study outlines a comprehensive outdoor play program that implements supportive policies, outdoor built environment change, professional development training and parent engagement across ECECs in the Greater Vancouver region of British Columbia Canada. Through a wait-list control cluster randomized trial study design, we found that children’s outdoor play behaviour demonstrated no consistent positive change following implementation of the PRO-ECO intervention. While positive effects were seen within some ECECs, the effect of the intervention was not sustained over time and was not consistent across all participating centres. Future studies interested in understanding children’s play behaviour change following an intervention should consider the multiple factors that influence the sustainability of interventions implemented in ECECs, including staff turnover, environment maintenance and the influence of adverse weather conditions.

The PRO-ECO study collected quantitative and qualitative data on educators’ perspectives and practices, children’s well-being and change in outdoor environment based on the Seven C’s design principles. Future analyses will seek to understand how these data illuminate the results for the primary outcome on children’s play participation to further understand the intervention uptake and effectiveness. Further analysis of secondary outcomes that are part of the PRO-ECO study will provide additional insights into children’s play behaviour, as well as impacts on their health, development and well-being, as a result of the PRO-ECO intervention. In addition, we anticipate that the qualitative data collected with ECEs as part of this study will provide further context on the absence of significant change in play versus non-play behaviour deducted within this analysis. The collective results from the PRO-ECO study will inform future expansion of the PRO-ECO intervention to additional ECECs and provide insightful alignment with ongoing international research on outdoor play in ECECs.

## Electronic supplementary material

Below is the link to the electronic supplementary material.

Supplementary Material 1

Supplementary Material 2

## Data Availability

All data, password-protected and stored in the secure network at the British Columbia Children’s Hospital Research Institute, will be available from MB upon reasonable request within 5 years of the completion of the study.
