# Supplementary material for: Results from the PROmoting Early Childhood Outside cluster randomized trial evaluating an outdoor play intervention in early childhood education centres
Source: Sci Rep. 2025 Jan 11;15:1713. doi: 10.1038/s41598-025-85397-1 (PMC11724985; doi:10.1038/s41598-025-85397-1)
Supplement: Supplementary file 1 — Supplementary Material 1 [file 41598_2025_85397_MOESM1_ESM.docx]

## Supplementary Material 2: PRO-ECO Play/Non-play Variable Rules

### Rules for coding non-play behaviour within the primary outcome variable (play/non-play):

- Child is wandering or transitioning between or within spaces with no clear engagement in any activity.
- Asking for help or needing assistance (and vocalizing this).
- Onlooking and deciding what to do before transitioning to other space/activity.
- Transitioning to another activity or space and not purposefully engaged in an activity; this can include running, walking or skipping to transition to another activity or space and is distinctly different from purposeful physical play.
- Eating while talking to other children/educator.
- Restorative play codes (Play Type 1, 2 or 3) on their own OR paired with a non-play code: Categorized into **Non-Play** for primary outcome variable;
- Within primary variable analysis (Play vs. Non-play), **Non-play** (in either Play Type 1 or 2) will ‘trump’ another play type (Physical, Exploratory, Restorative, etc.) in this dichotomization.
- If a child is preparing to eat, such as sitting down and getting out their lunch, but not actually eating yet = Non-play ‘Other’

### Rules for coding play behaviour within primary outcome variable (play/non-play)

- Child transitions into a new space, but is engaged in an activity for the duration of the time.
- Restorative play codes paired with another play code (physical, exploratory, imaginative, play with rules, bio and expressive)

**Non-play: Transition**

**Non-play: Nutrition**

**Restorative Play: Onlooking**

**Non-play: Aggression & Distress**

**Non-play: Aggression**

Is the child participating in a non-playful, physical interaction?

Is the child upset, crying or sad?

**Non-play: Distress**

Is the child eating or drinking?

Is the child talking to a friend or teacher while eating?

**Non-play: Nutrition**

Is the child resting?

Is the child stationary?

Is the child watching other children or an activity take place?

**Non-play: Nutrition**

Is the child engaged in an activity?

**Non-play: Transition**

**This is most likely a PLAY activity**

*(refer to TOPO)*

Is the child dis-engaging from an activity?

*(e.g. determining what to do next)*

Is the child moving to another space?

**Restorative Play: Resting**

**Restorative Play: Retreat**

Is the child cleaning up or taking care of themselves?

Is the child retreating in an enclosed space away from other children?

Is the child taking care of themselves or another child?

*(e.g. tying shoes, zipping jacket, hand wash)*

**Non-play: Self-care**

**Non-play: Other**

Is the child exhibiting aggression or distress?
