# Supplementary material for: Results from the PROmoting Early Childhood Outside cluster randomized trial evaluating an outdoor play intervention in early childhood education centres
Source: Sci Rep. 2025 Jan 11;15:1713. doi: 10.1038/s41598-025-85397-1 (PMC11724985; doi:10.1038/s41598-025-85397-1)
Supplement: Supplementary file 2 — Supplementary Material 2 [file 41598_2025_85397_MOESM2_ESM.pdf]

# Supplementary Material 1: PRO-ECO Intervention Details by Study ECEC Site

## Loose parts implemented across all ECECs

- Milk crates
- Popsicle sticks
- Buttons
- Mason jar bangles
- Pillow cases
- Fabric
- Ribbons
- Shells
- Tactile objects
- Kitchen items
- Piping

## Intervention components specific to each ECEC

*Centre A*

Built Environment Modifications

- Wooden deck tiles
- Wooden puzzle blocks
- Shade net with gourds, gourd chimes
- Ceramic planters and plantings
- Netting over play structure
- Additional garden bed plantings and replacement of dead tree

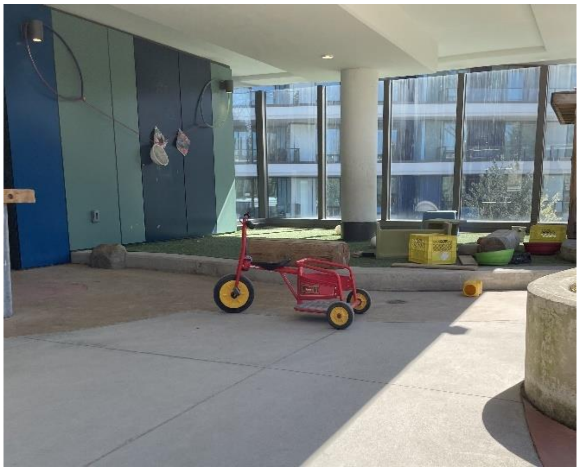

Figure S1: Centre A Open Space Pre-Intervention

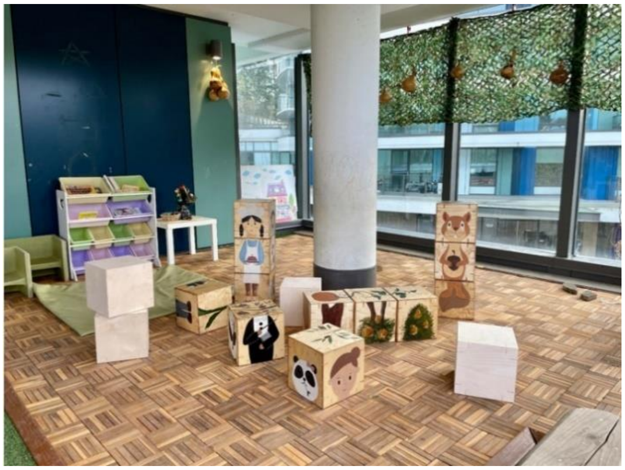

Figure S2: Centre A Open Space Post-Intervention

*Centre B*

Built Environment Modifications

- Shade sail
- Planter bags, planter boxes, plantings
- Expanded sandbox steps
- Rocking rainbows and water troughs with stands and wooden balls
- Water hoses and mulching

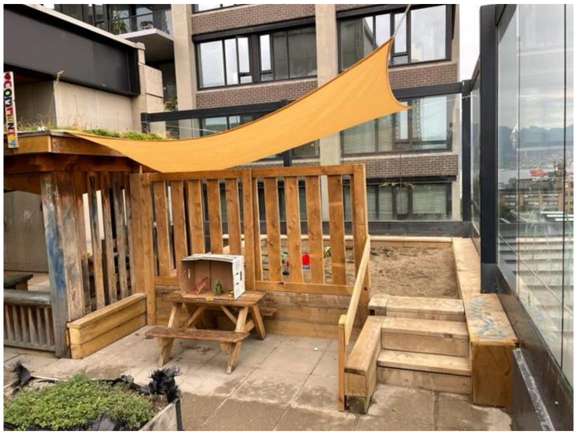

Figure S3: Centre B Sandbox Pre-Intervention

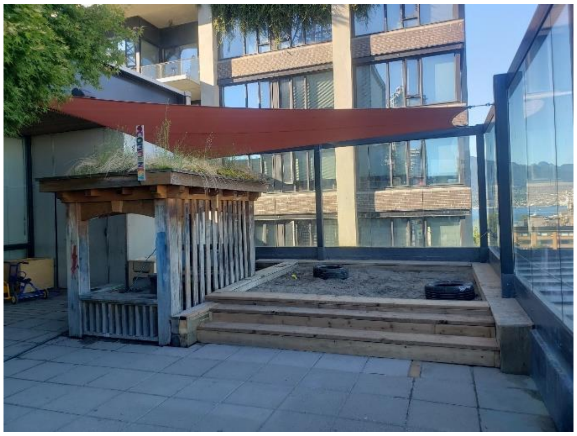

Figure S4: Centre B Sandbox Post-Intervention

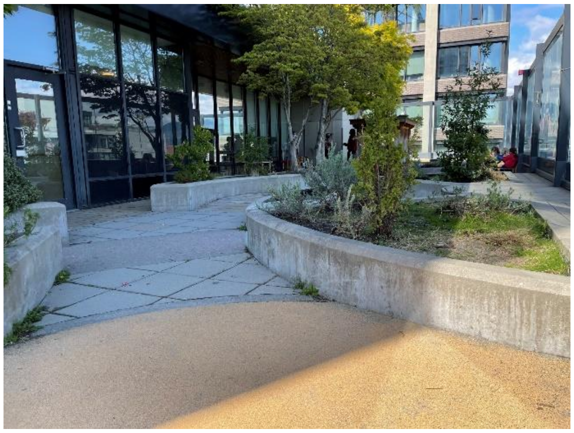

Figure S5: Centre B Gardening Pre-Intervention

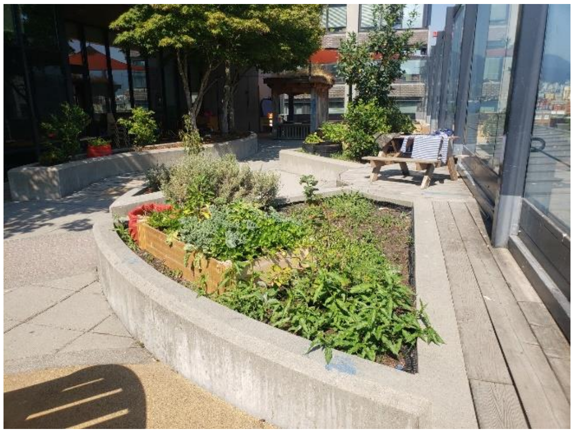

Figure S6: Centre B Gardening Post-Intervention

*Centre C*

Built Environment Modifications

- Shade sail
- Planter bags
- Ribbon arbor
- Wood climber
- Labyrinth (flagstones and grass)

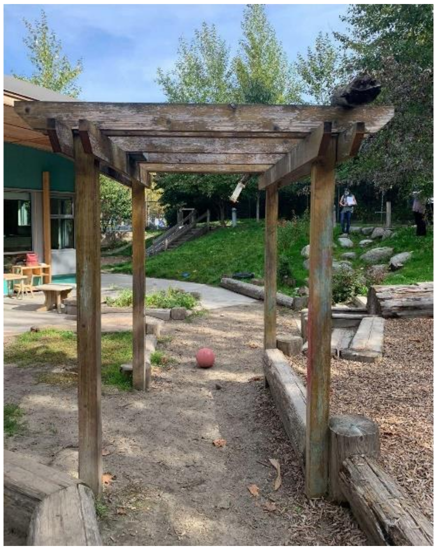
Figure S7: Centre C Ribbon Arbor Pre-Intervention

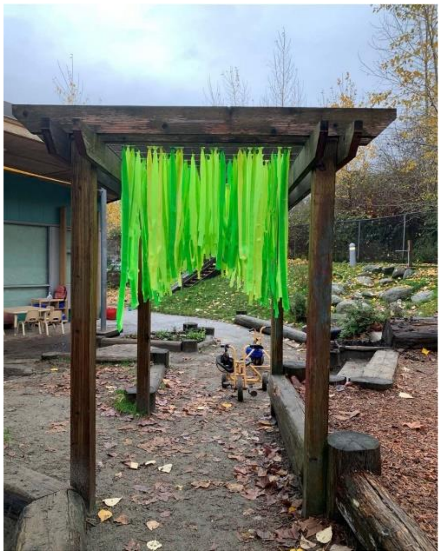
Figure S8: Centre C Ribbon Arbor Post-Intervention

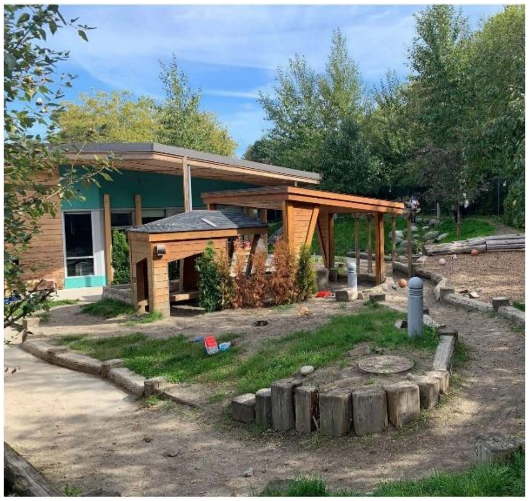
Figure S9: Centre C Labyrinth Pre-Intervention

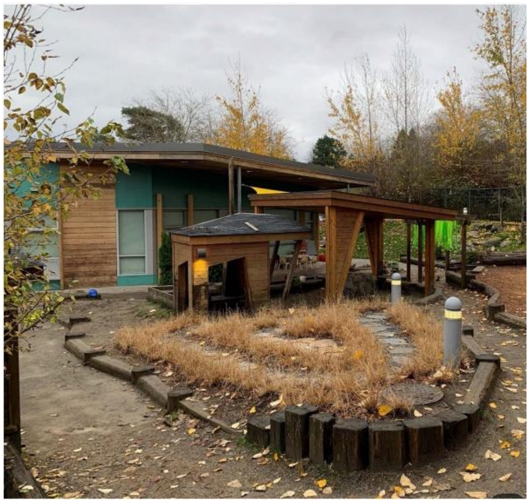
Figure S10: Centre C Labyrinth Post-Intervention

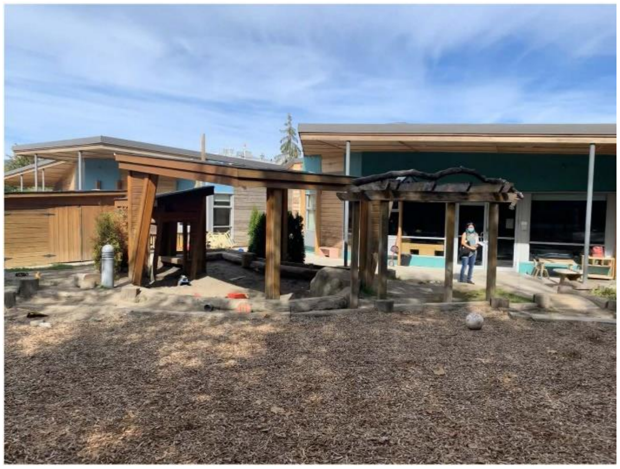
Figure S11: Centre C Open Space Pre-Intervention

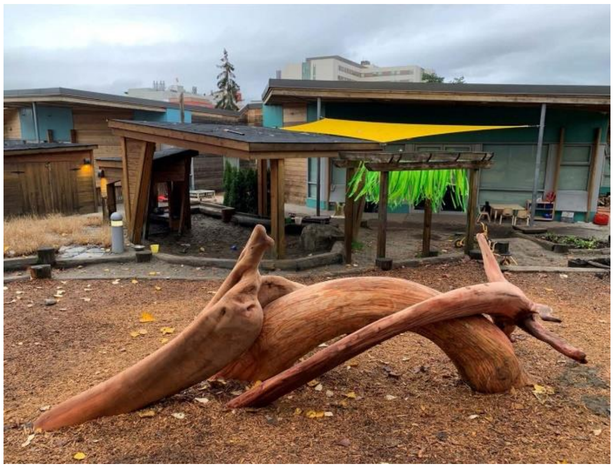
Figure S12: Centre C Open Space Post- Intervention

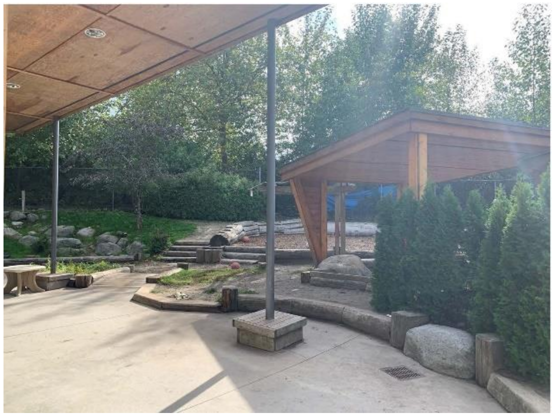

Figure S13: Centre C Shade Sail Pre-Intervention

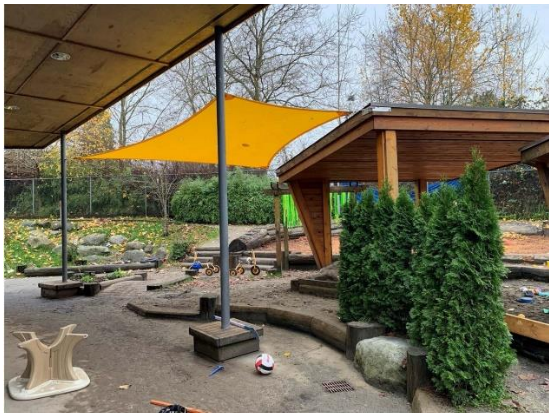

Figure S14: Centre C Shade Sail Post-Intervention

*Centre D*

Built Environment Modifications

- Plantings and tree cookies
- Water wall and rocky bamboo water area
- Shade sails and sandbox shade cover

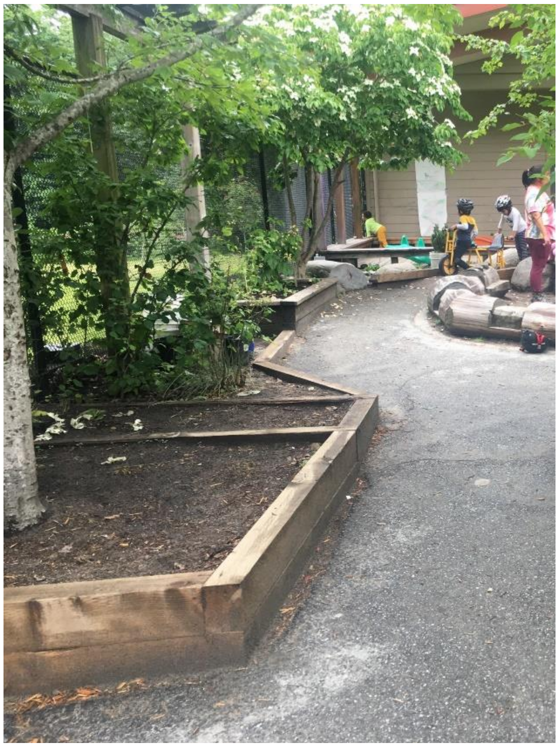

Figure S15: Centre D Gardening Area Pre-Intervention

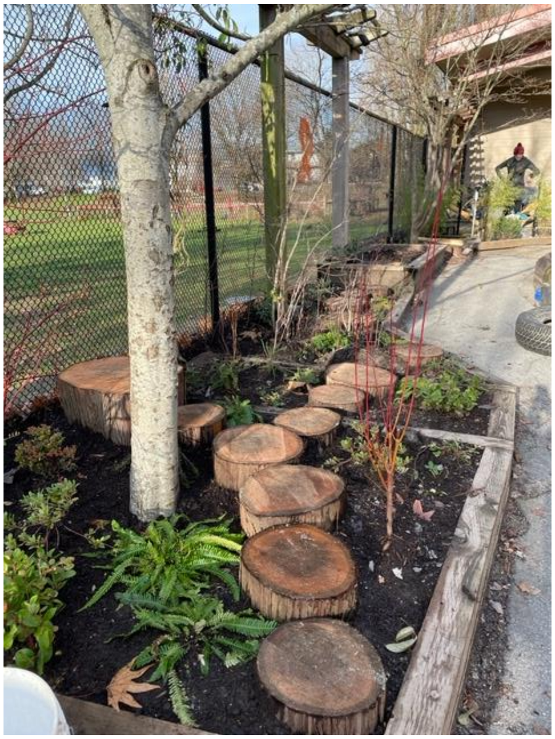

Figure S16: Centre D Gardening Area Post-Intervention

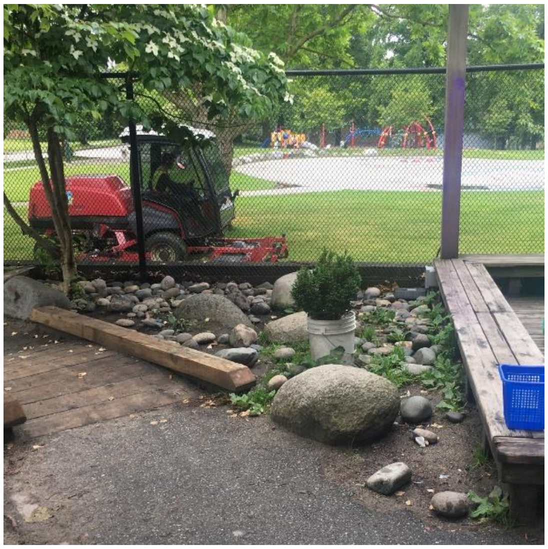
Figure S17: Centre D Rock Area Pre-Intervention

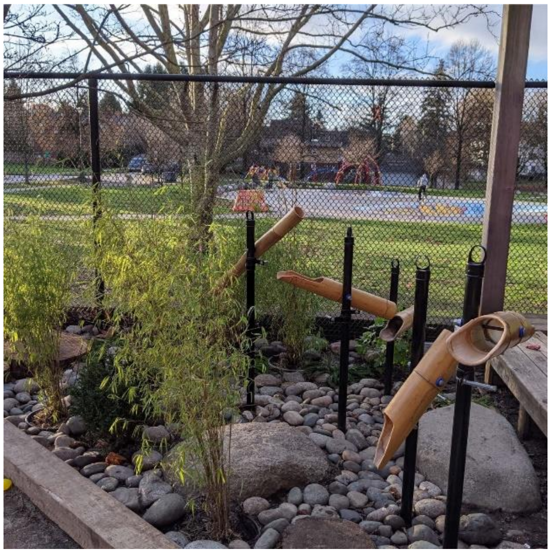
Figure S18: Centre D Rock Area Post-Intervention

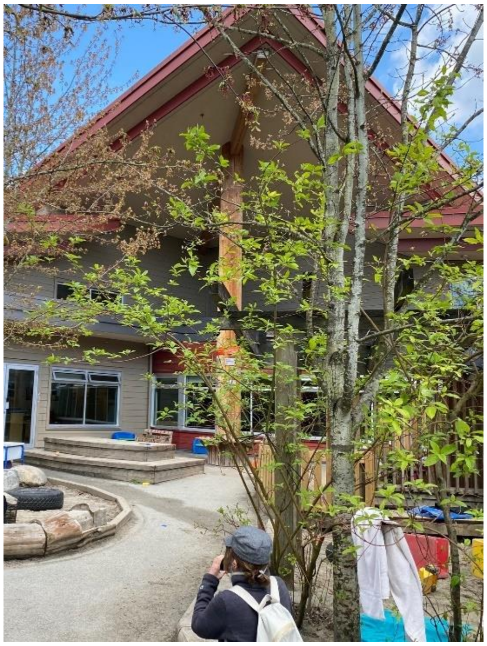
Figure S19: Centre D Covered Area Pre-Intervention

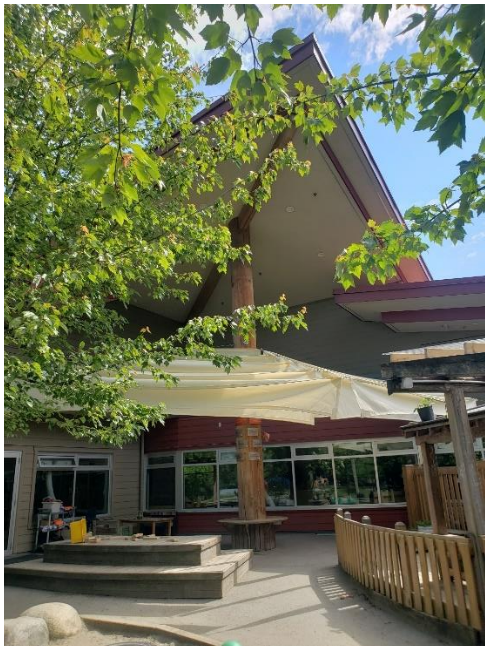
Figure S20: Centre D Covered Area Post-Intervention

*Centre E*

Built Environment Modifications

- Shade sail
- Expanded fenced area
- Planter bags and plantings
- Hanging ribbon
- Water troughs with stands and wooden balls
- Water hose

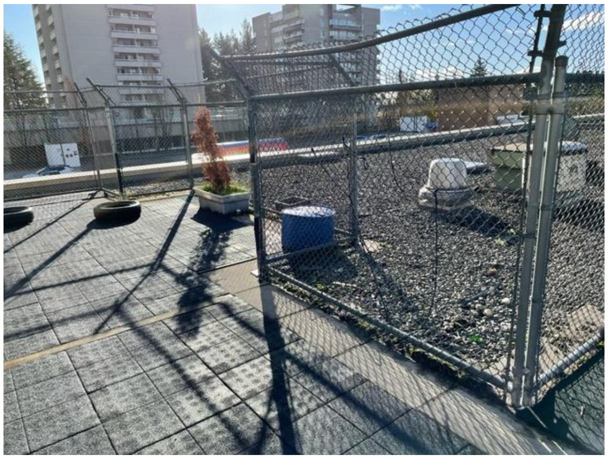

Figure S21: Centre E Fence Line Pre-Intervention

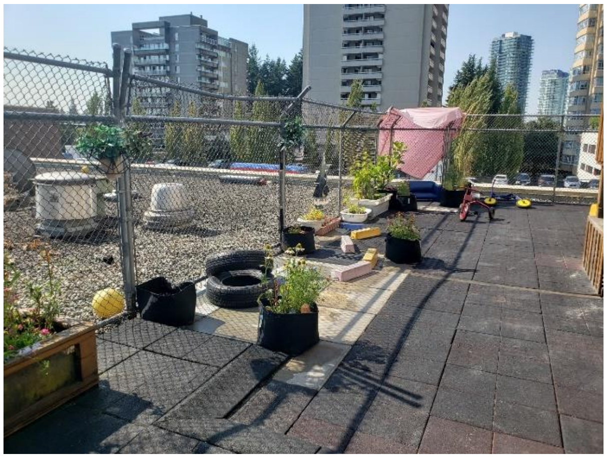

Figure S22: Centre E Fence Line Post-Intervention

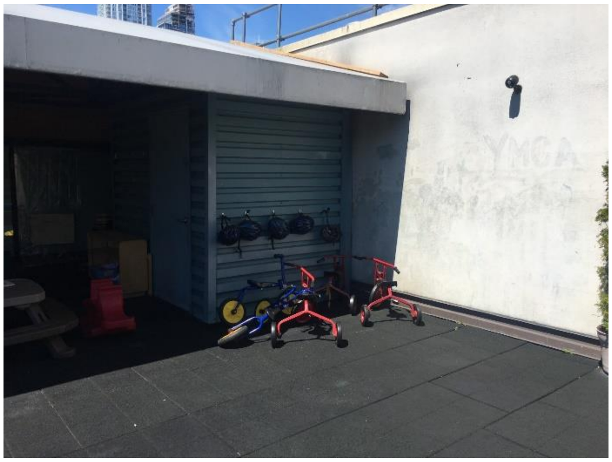

Figure S23: Centre E Corner Space Pre-Intervention

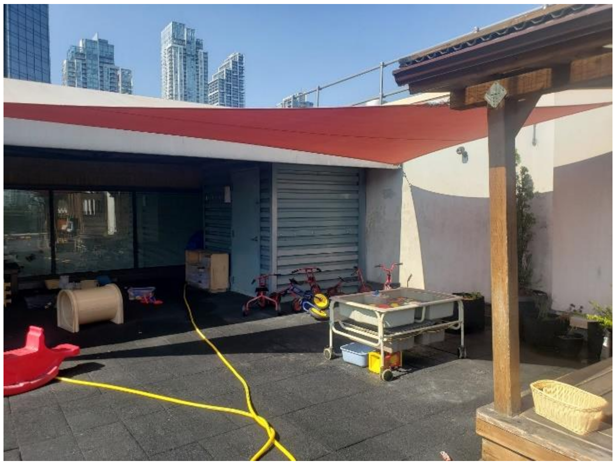

Figure S24: Centre E Corner Space Post-Intervention

*Centre F*

Built Environment Modifications

- Plantings and planter bags
- Windsock fish
- Rocking rainbows and pickler arch and balance boards
- Water troughs with stands and wooden balls
- Water hose

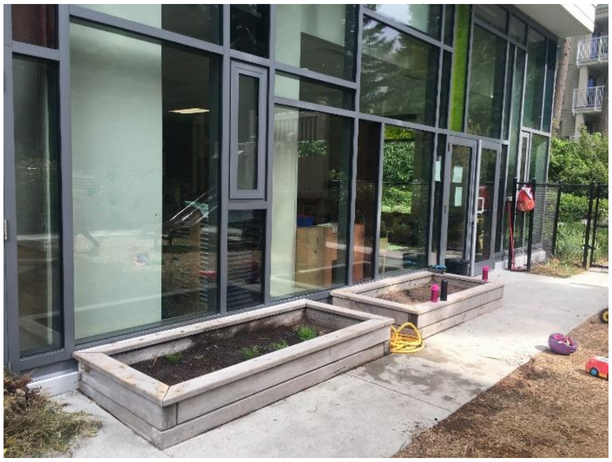

Figure S25: Centre F Gardening Area Pre-Intervention

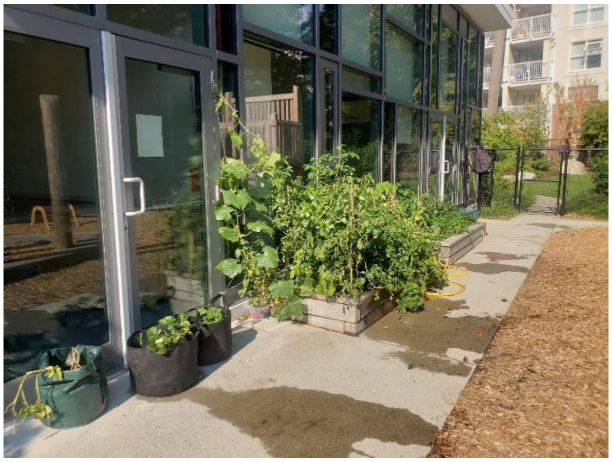

Figure S26: Centre F Gardening Area Post-Intervention

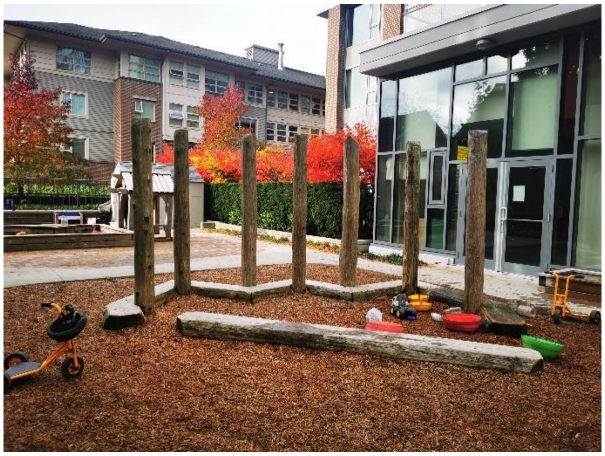

Figure S27: Centre F Wood Posts Pre-Intervention

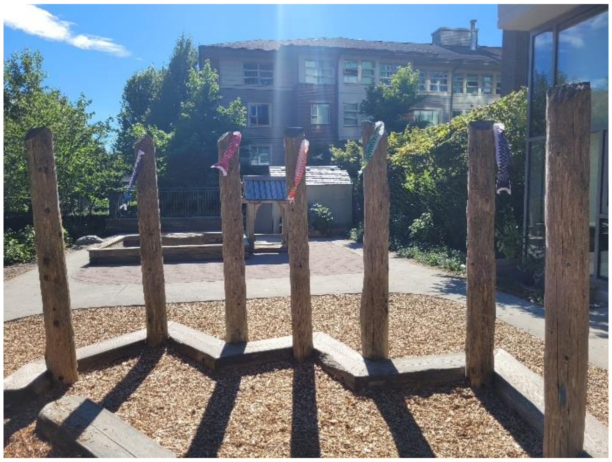

Figure S28: Centre F Wood Posts Post-Intervention

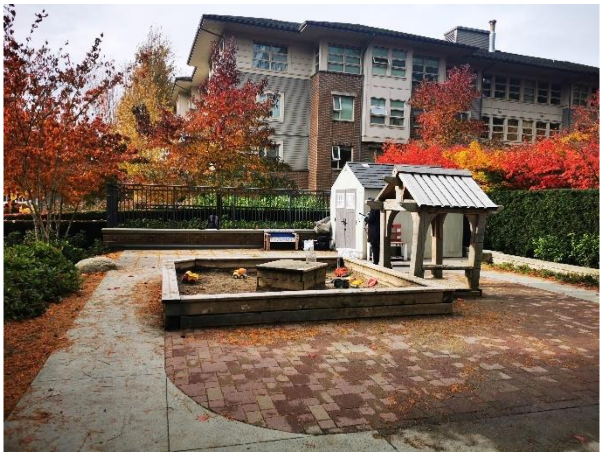

Figure S29: Centre F Open Space Pre-Intervention

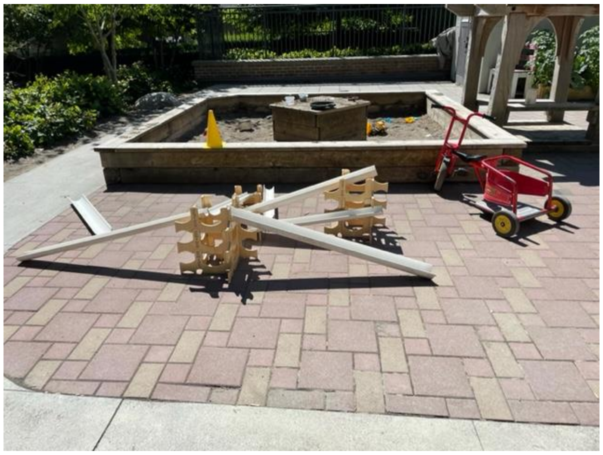

Figure S30: Centre F Open Space Post-Intervention

*Centre G*

Built Environment Modifications

- Fence banners and rainbow weaving
- Wind chimes

- Water pump, large wooden bucket and water troughs
- Grass corridor (plantings) with ferns and fairy pebbles

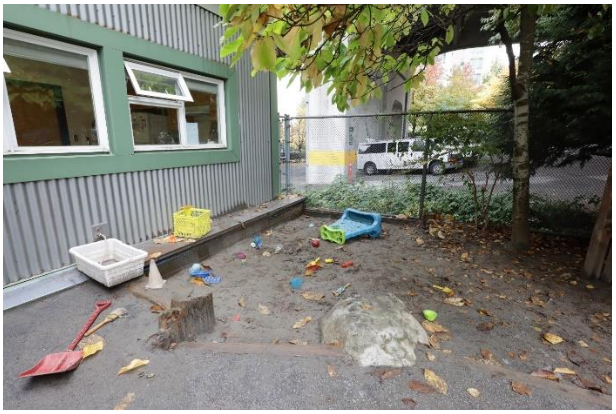

Figure S31: Centre G Sandbox Area Pre-Intervention

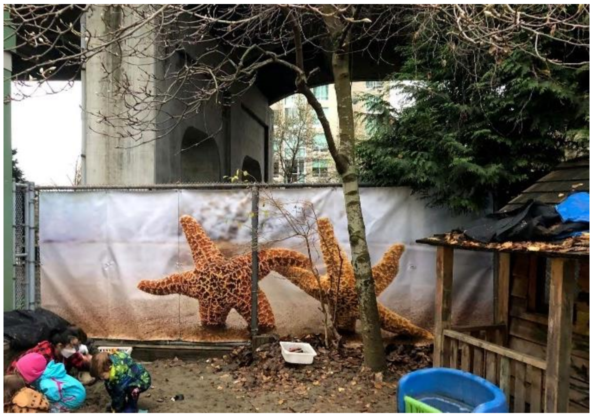

Figure S32: Centre G Sandbox Area Post-Intervention

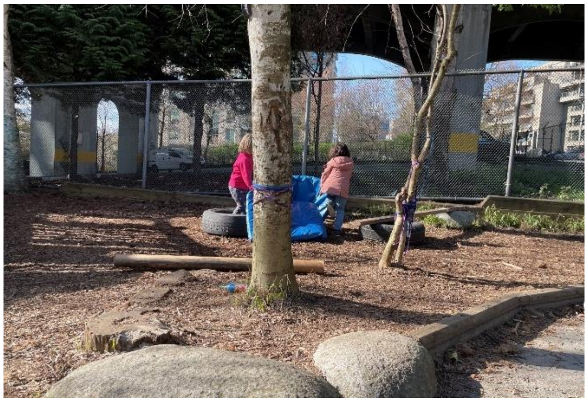

Figure S33: Centre G Open Area Pre-Intervention

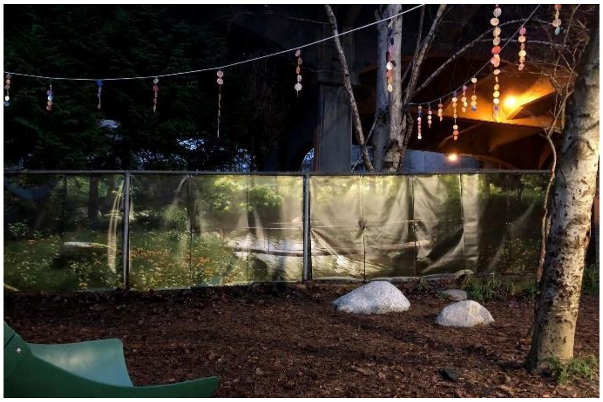

Figure S34: Centre G Open Area Post-Intervention

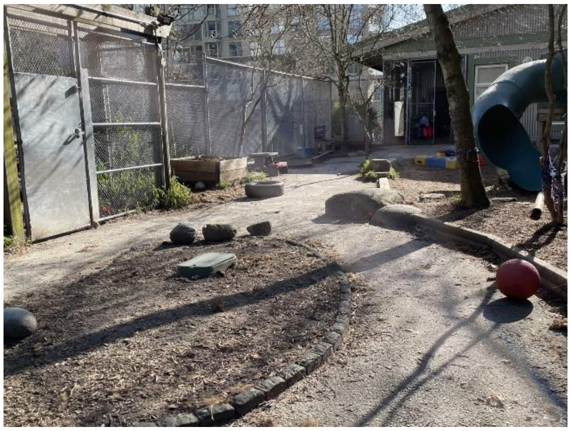

Figure S35: Centre G Outdoor Entrance Pre-Intervention

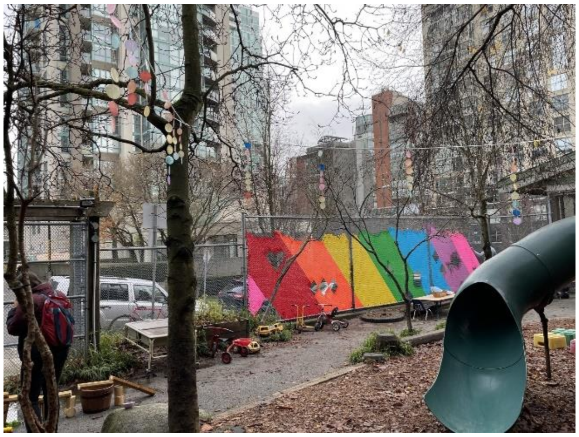

Figure S36: Centre G Outdoor Entrance Post-Intervention

*Centre H*

Built Environment Modifications

- Labyrinth (pathway, rocks, tree)
- Planter bags and planter boxes with plantings

- Ribbon grids
- Shade sail
- Rocking rainbows, pickler arch and balance boards
- Water troughs with stands and wooden balls
- Hose and hose bib

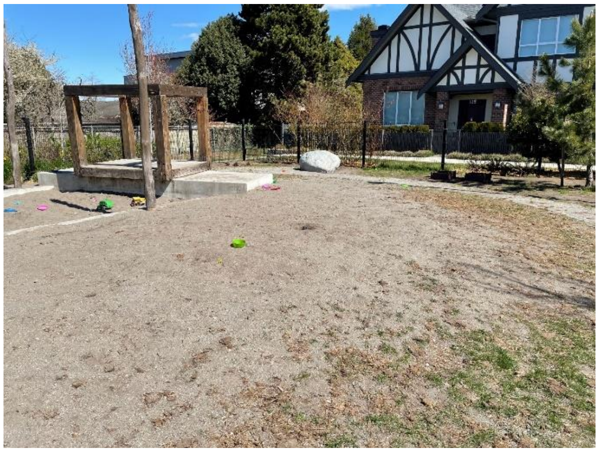

Figure S37: Centre H Open Space Pre-Intervention

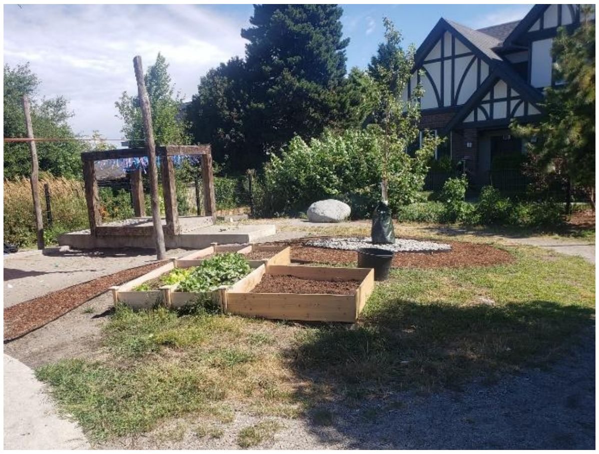

Figure S38: Centre H Open Space Post-Intervention

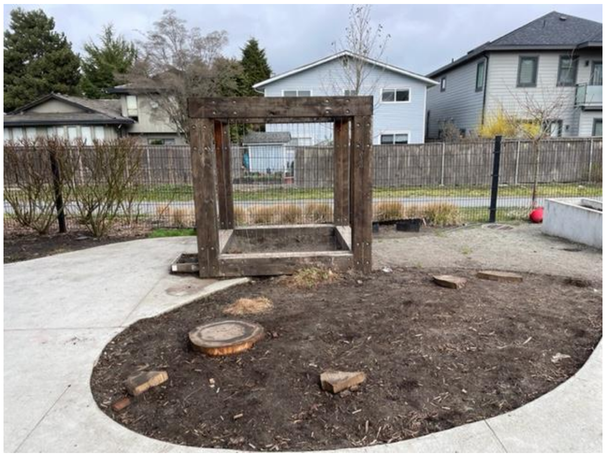

Figure S39: Centre H Garden Area Post-Intervention

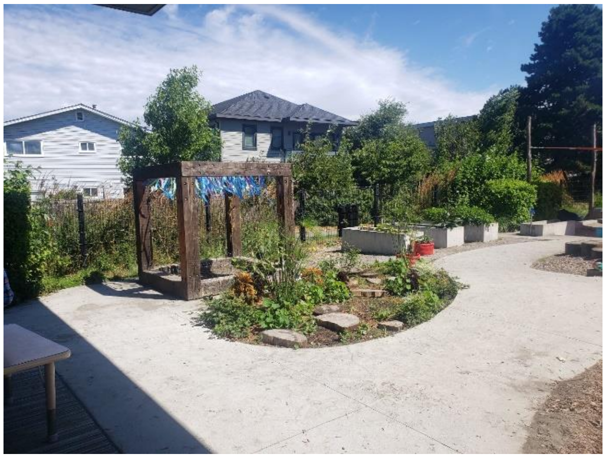

Figure S40: Centre H Garden Area Post-Intervention
